# Distribution of Mixotrophy and Desiccation Survival Mechanisms across Microbial Genomes in an Arid Biological Soil Crust Community

**DOI:** 10.1128/mSystems.00786-20

**Published:** 2021-01-12

**Authors:** Dimitri V. Meier, Stefanie Imminger, Osnat Gillor, Dagmar Woebken

**Affiliations:** a Division of Microbial Ecology, Department of Microbiology and Ecosystem Science, Centre for Microbiology and Environmental Systems Science, University of Vienna, Vienna, Austria; b Zuckerberg Institute for Water Research, Blaustein Institutes for Desert Research, Ben Gurion University of the Negev, Sde Boker, Israel; MIT

**Keywords:** biological soil crust, dormancy, metagenomics, mixotrophy, survival

## Abstract

Desert surface soils devoid of plant cover are populated by a variety of microorganisms, many with yet unresolved physiologies and lifestyles. Nevertheless, a common feature vital for these microorganisms inhabiting arid soils is their ability to survive long drought periods and reactivate rapidly in rare incidents of rain. Chemolithotrophic processes such as oxidation of atmospheric hydrogen and carbon monoxide are suggested to be a widespread energy source to support dormancy and resuscitation in desert soil microorganisms. Here, we assessed the distribution of chemolithotrophic, phototrophic, and desiccation-related metabolic potential among microbial populations in arid biological soil crusts (BSCs) from the Negev Desert, Israel, via population-resolved metagenomic analysis. While the potential to utilize light and atmospheric hydrogen as additional energy sources was widespread, carbon monoxide oxidation was less common than expected. The ability to utilize continuously available energy sources might decrease the dependency of mixotrophic populations on organic storage compounds and carbon provided by the BSC-founding cyanobacteria. Several populations from five different phyla besides the cyanobacteria encoded CO_2_ fixation potential, indicating further potential independence from photoautotrophs. However, we also found population genomes with a strictly heterotrophic genetic repertoire. The highly abundant *Rubrobacteraceae* (*Actinobacteriota*) genomes showed particular specialization for this extreme habitat, different from their closest cultured relatives. Besides the ability to use light and hydrogen as energy sources, they encoded extensive O_2_ stress protection and unique DNA repair potential. The uncovered differences in metabolic potential between individual, co-occurring microbial populations enable predictions of their ecological niches and generation of hypotheses on the dynamics and interactions among them.

**IMPORTANCE** This study represents a comprehensive community-wide genome-centered metagenome analysis of biological soil crust (BSC) communities in arid environments, providing insights into the distribution of genes encoding different energy generation mechanisms, as well as survival strategies, among populations in an arid soil ecosystem. It reveals the metabolic potential of several uncultured and previously unsequenced microbial genera, families, and orders, as well as differences in the metabolic potential between the most abundant BSC populations and their cultured relatives, highlighting once more the danger of inferring function on the basis of taxonomy. Assigning functional potential to individual populations allows for the generation of hypotheses on trophic interactions and activity patterns in arid soil microbial communities and represents the basis for future resuscitation and activity studies of the system, e.g., involving metatranscriptomics.

## Abstract (precis)

This study represents a comprehensive community-wide genome-centered metagenome analysis of biological soil crust (BSC) communities in arid environments, providing insights into the distribution of genes encoding different energy generation mechanisms, as well as survival strategies, among populations in an arid soil ecosystem. It reveals the metabolic potential of several uncultured and previously unsequenced microbial genera, families, and orders, as well as differences in the metabolic potential between the most abundant BSC populations and their cultured relatives, highlighting once more the danger of inferring function on the basis of taxonomy.

## INTRODUCTION

Drylands constitute more than 35% of land surface and are currently expanding (1). They experience prolonged periods of drought interrupted by brief rain events, which limit vascular plant growth. Yet these environments are not devoid of life (2, 3). Dryland soils are inhabited by diverse microbial communities whose members are assumed to endure extended droughts in a largely inactive state often called “dormancy” but reactivate quickly once hydrated. Although the definitions of dormancy differ (reviewed in references 4 and 5), typical examples include sporulation performed by Gram-positive *Firmicutes* and some *Actinobacteriota* (6, 7), cysts with enforced cell envelopes formed by some *Alphaproteobacteria* (8), and specialized dormant cells termed akinetes formed by cyanobacteria of the orders *Nostocales* and *Stigonematales* (9). Yet, dryland soils are inhabited by a variety of other microbial clades (3, 10) whose mechanisms of surviving desiccation are unknown. It was recently suggested that many soil microorganisms might rely on inorganic energy sources such as light and oxidation of atmospheric trace gases (e.g., dihydrogen [H_2_] and carbon monoxide [CO]) to maintain the cell’s integrity in a dormant state (11). Furthermore, trace gas oxidation could potentially support primary production, as oxidation of atmospheric H_2_ has been shown to sustain CO_2_ fixation in an Antarctic desert soil (12). However, it is still unclear if these processes are present in other arid ecosystems and the extent of their distribution among the microbial populations within a given soil community, especially in taxonomic groups without cultured representatives.

The aim of this study was to assess the distribution of potential physiologies regarding carbon (C) metabolism, energy generation, and dormancy mechanisms across different populations of a dryland soil microbial community. We performed population-resolved metagenomics analysis of microbiota inhabiting biological soil crusts (BSCs) from the Negev Desert, Israel. BSCs cover the topmost layer of many arid soils and are formed by highly adapted microbial communities (1). Arid and hyperarid BSCs are characterized by the presence of filamentous cyanobacteria, which secrete ample extracellular polymeric substances (EPS) crucial for the structure and integrity of the crust (1, 13) and supply organic C to heterotrophic community members (14, 15). Their primary production and matrix secretion provide an optimized environment for soil microorganisms, resulting in significantly increased microbial abundance and diversity in BSC in comparison to that of the soil beneath (16, 17). In addition, BSC microbial communities are essential to this ecosystem, as they take over important ecological functions such as CO_2_ and N_2_ fixation and prevent the erosion of underlying soil (1, 18). Being populated by dryland soil microbiota yet containing more biomass than barren soil (10, 16) makes BSCs a good model system for *in situ* studies on microbial dormancy and resuscitation strategies.

Most of our functional knowledge on BSC native taxa stems from cultivation and gene amplification studies targeting specific metabolisms, such as nitrification (19), N_2_ fixation (20, 21), methanogenesis (22), or anoxygenic photosynthesis (23). However, gene amplification studies do not provide information on the abundances and metabolisms of other co-existing microbial community members. More recent metagenomic studies of soil crusts, or desert soils in general, identified broader categories of potential metabolic functions present in these communities (24–31). However, as these investigations were done mostly without assembly or genome binning, taxonomic assignments of the identified genomic potential were largely missing. Neither bulk metagenomics nor functional gene-centered studies can determine if, e.g., two metabolisms are performed by the same species or if two steps of a metabolism are divided between different species. Yet, gaining information on the potential metabolism of individual, co-occurring microbial populations is essential for predicting their ecological niches and for generating hypotheses on the dynamics and interactions among members of this BSC ecosystem. With the population genomes at hand, such hypotheses can now be tested by targeting specific microbial groups and their activities, for instance by employing transcriptomic investigations.

## RESULTS

### Microbial community composition.

**FIG 1:**
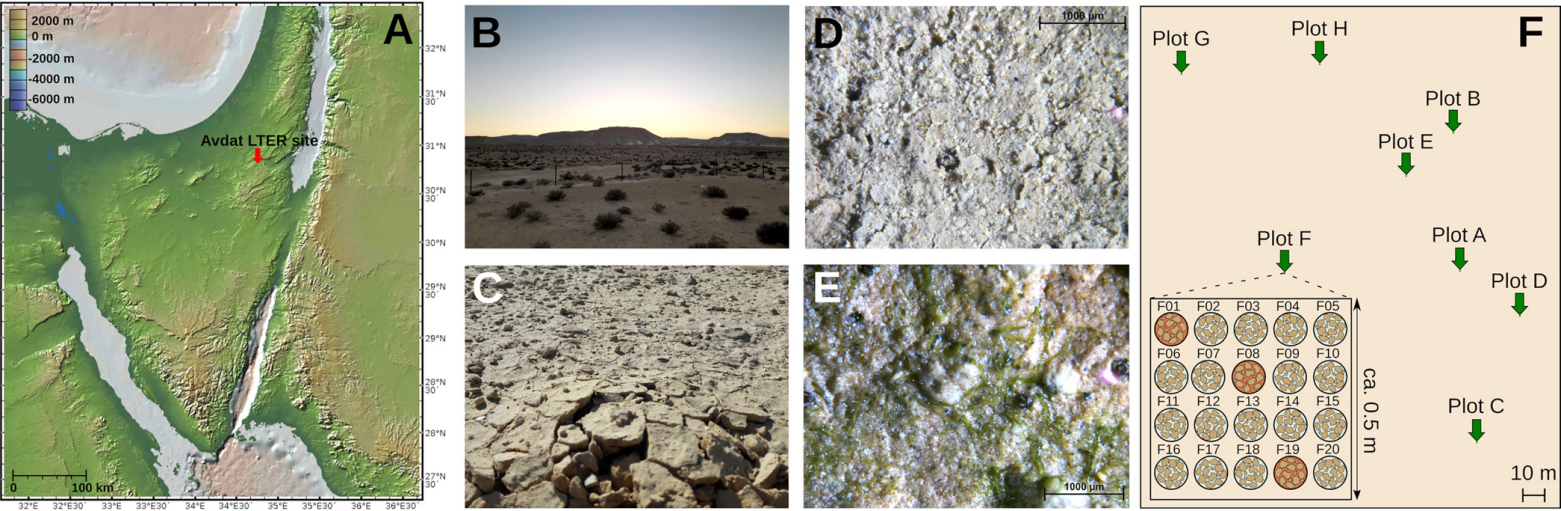
Location of and sampling at the long-term ecological research (LTER) site in the Negev Desert, Israel. (A) Location of the Avdat LTER site. The map was generated with GeoMapApp and Global Multi-Resolution Topography data (http://www.geomapapp.org)/CC BY/CC BY (Ryan et al., 2009 [130]). (B) Landscape at the LTER site. Crust was sampled from shrub-free patches with a homogeneous surface appearance. (C) Close-up of soil crust pieces. The crusts were light in color and largely free of lichens or mosses. Coherent pieces of soil crust of ca. 5 mm thickness were collected and transported in petri dishes. (D) Surface of dry crust as seen through a stereoscope. (E) Surface of a rehydrated crust piece after 24 h as seen through a stereoscope. Large bundles of *Cyanobacteria* could be observed on the surface of soil crusts starting at around 2 h and reaching the largest extent by 12 h. (F) Areal distribution of sampling plots. From each plot of ca. 0.5 by 0.5 m, 20 petri dishes of crust pieces were sampled. For 16S rRNA amplicon sequencing, crust pieces were randomly chosen from three petri dishes per plot, as is exemplified for plot F.

**FIG 2:**
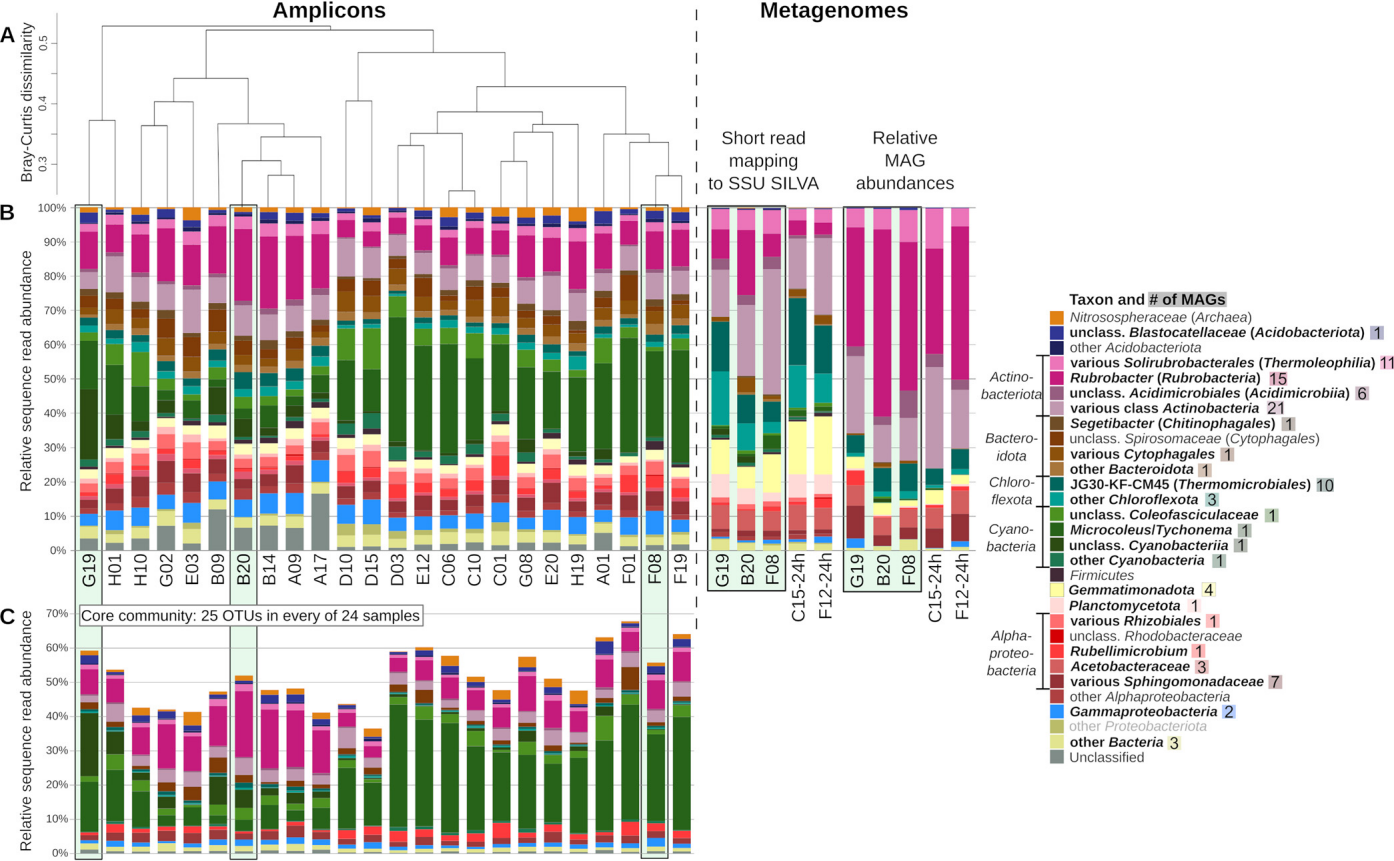
Microbial community composition of the biological soil crusts at Avdat LTER, Negev Desert, Israel. (A) Hierarchical clustering of samples based on the Bray-Curtis dissimilarity of the microbial community compositions at the Swarm-OTU level. (B) Taxonomic composition determined by amplicon sequencing of 16S rRNA gene fragments, unassembled metagenomic reads mapping to SILVA SSU database, and read mapping to GTDB-classified population genomes. Taxa represented by MAGs are highlighted in bold in the legend, and the number of corresponding MAGs is indicated next to the taxon name. (C) Relative sequence abundances of 25 OTUs present in every sample. The letters A to H in the sample names refer to the different sampling plots illustrated in Fig. 1F, while numbers indicate the randomly chosen subsamples (petri dishes) per plot. Outlined samples highlight different data originating from the very same DNA extracts (G19, B20, F08). Classification and relative abundances of OTUs and detailed composition of unassembled metagenomes based on read mapping to the SILVA database can be found in Data Set S1 in the supplemental material.

Being collected from an arid site with less than 100 mm of annual rainfall (32), the samples represented light-colored *Cyanobacteria*-dominated BSCs (Fig. 1). Before choosing samples for metagenomic sequencing, we assessed the diversity and spatial heterogeneity of the BSC microbial communities at the Avdat long-term ecological research (LTER) site (Fig. 1) by sequencing 16S rRNA gene amplicons from 24 samples originating from eight different sampling positions labeled A to H within the LTER site (Fig. 1; see library statistics in Table S1 in the supplemental material). After quality filtering, denoising, and determination of amplicon sequence variants (ASVs), the average sequence count was 9,831 ± 3,690 reads per sample (Table S1). The 1,056 ASVs clustered into 921 operational taxonomic units (OTUs) via Swarm percentage identity-independent single-linkage clustering (33). The composition of BSC microbial communities appeared homogeneous across the site (Fig. 2). Between 37% and 68% of reads in any sample were attributed to the same ubiquitous 25 OTUs and thus represented taxa shared among all samples (Fig. 2C). An additional 17% to 35% of reads per sample belonged to 103 OTUs that were detected in more than half, but not all, samples. As expected, the community was composed of large proportions of *Cyanobacteria* and *Actinobacteriota* and smaller fractions of *Alphaproteobacteria*, *Bacteroidota*, *Chloroflexota*, *Gemmatimonadota*, and *Acidobacteriota*.

10.1128/mSystems.00786-20.8 TABLE S1 16S rRNA gene amplicon sequencing metrics. The table provides the numbers of quality-filtered reads obtained from each sample, the numbers of ASVs and Swarm-generated OTUs detected in each sample (richness), and the percentages of reads assigned to ubiquitous OTUs, OTUs appearing in more than 50% of the samples, and OTUs appearing only in fewer than four samples. Download Table S1, PDF file, 0.03 MB. Copyright © 2021 Meier et al. 2021 Meier et al. This content is distributed under the terms of the Creative Commons Attribution 4.0 International license.

10.1128/mSystems.00786-20.1 DATA SET S1 Large spreadsheet providing relative abundances and taxonomic classification of Swarm OTUs as well as relative abundances of microbial genera as determined by short read mapping to SILVA SSU132 database with PhyloFlash v.30 (13). Only genera with three or more reads mapping were considered as detected. Download Data Set S1, XLSX file, 0.2 MB. Copyright © 2021 Meier et al. 2021 Meier et al. This content is distributed under the terms of the Creative Commons Attribution 4.0 International license.

Analysis of our metagenome libraries (see metrics in Table S2) with Nonpareil (34) indicated that by sequencing between 16 and 32 Gbp per sample, we covered between approximately 56% (sample C15) and 74% (sample B20) of the diversity. If taken together, the libraries covered 79% of the diversity. The Nonpareil diversity index for our samples was between 20.7 and 21.9 (Table S2, part A), which is similar to that of Atacama top soils (21.2) (3), higher than that of human-associated (17.5 to 18) or lake water (19) microbiomes, but lower than that of cornfield soil (23) (all reported in reference 34). Interestingly, based on unassembled read mapping to the SILVA SSU132 database (Data Set S1), the relative abundance of *Cyanobacteria* in the metagenome was much lower than in the amplicon data, although the libraries were prepared from the same DNA extract. This phenomenon was reported previously in BSCs (24), and possible explanations are discussed in Text S2 in the supplemental material.

10.1128/mSystems.00786-20.3 TEXT S1 A detailed description of the metagenome analysis providing the reasoning behind the choice of methods and analysis steps performed. The scripts referenced in this section can be found in the following Github repository: https://github.com/meierdv/avdat_metagenome. Download Text S1, PDF file, 0.1 MB. Copyright © 2021 Meier et al. 2021 Meier et al. This content is distributed under the terms of the Creative Commons Attribution 4.0 International license.

10.1128/mSystems.00786-20.4 TEXT S2 Discussion of possible causes for differences in relative abundances of microbial taxa between 16S rRNA gene amplicon data and metagenomic sequencing data, with a focus on difference in relative abundance of *Cyanobacteria*. Download Text S2, PDF file, 0.04 MB. Copyright © 2021 Meier et al. 2021 Meier et al. This content is distributed under the terms of the Creative Commons Attribution 4.0 International license.

10.1128/mSystems.00786-20.9 TABLE S2 Metagenomic sequencing and assembly metrics. Part A provides the basic metrics of metagenomic sequencing efforts, such as sequencing depth, the estimation of genomic diversity covered (determined by Nonpareil), and percentages of reads represented by the assembly and analyzed MAGs. Part B provides the basic metrics of the initial metagenomic assembly. Download Table S2, PDF file, 0.04 MB. Copyright © 2021 Meier et al. 2021 Meier et al. This content is distributed under the terms of the Creative Commons Attribution 4.0 International license.

**FIG 3:**
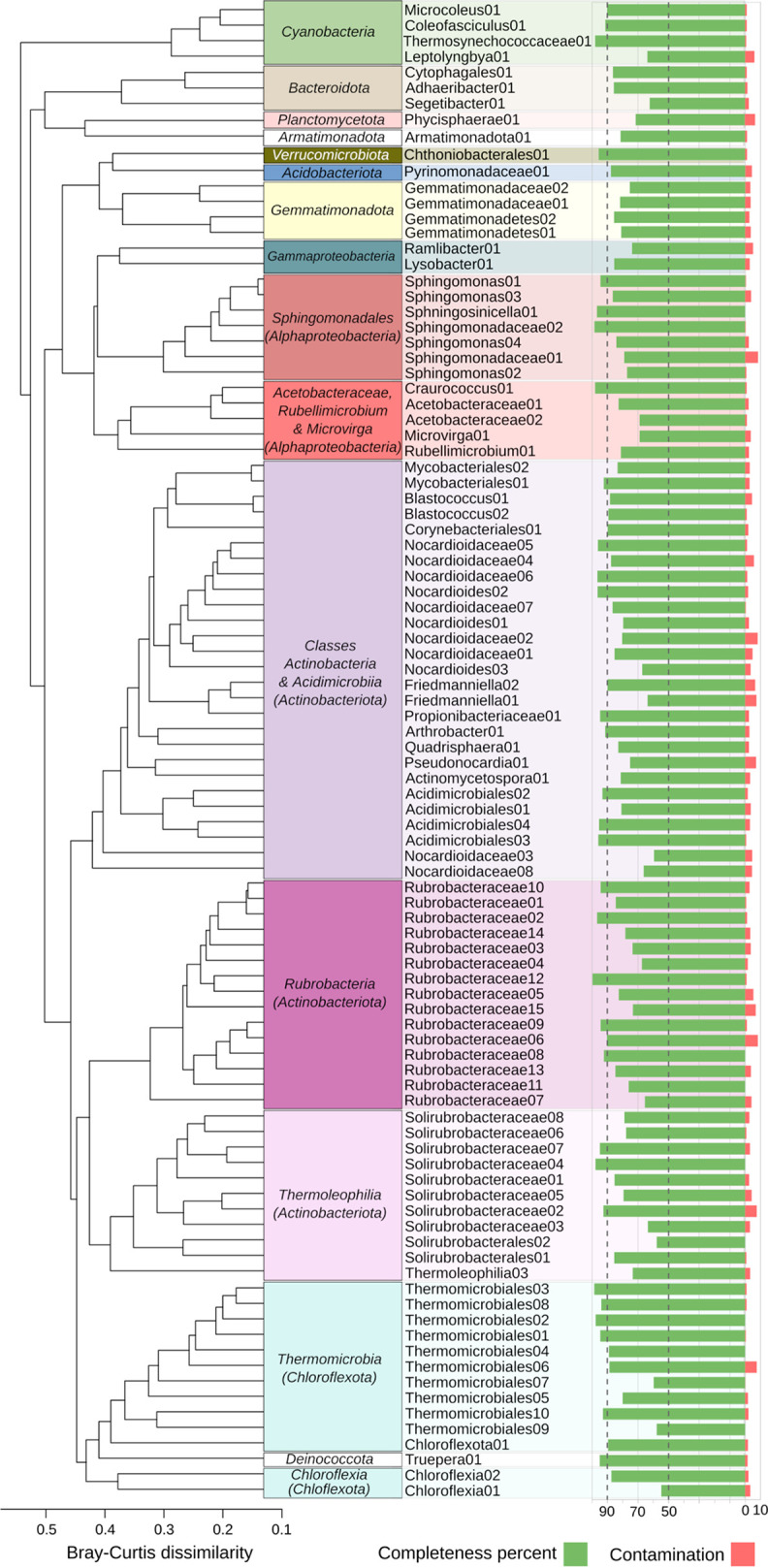
Clustering of the metagenome-assembled genomes (MAGs) based on presence-absence matrix of functions as assigned by EggNOGs. Different orthologous groups annotated with the same function were summarized into one functional category. In total, 8,279 functions were used for clustering. Functions appearing only in one MAG were removed from the matrix. Hierarchical clustering by average linkage was performed based on Bray-Curtis dissimilarity with the vegan package in R. Green bars indicate the percentage completeness of MAGs based on the presence of lineage-specific essential single-copy genes; red bars indicate the percentage of single-copy genes present in several different copies (according to CheckM).

From sequence reads of five crust samples, we obtained 96 metagenome-assembled population genomes (MAGs) (main metrics in Table S3), representing most of the microbial taxa that we detected in the unassembled data (Fig. 2, short read mapping; Data Set S1) and 24% to 36% of the raw reads (mapped with identity of  >95%). According to Genome Taxonomy Database classification (35, 36) (Table S3, GTDB classification data, including average nucleotide identities [ANIs]) and our phylogenetic analysis (Fig. S1), all 96 MAGs represented a previously unsequenced species, 59 MAGs belonged to 32 novel genera, 13 represented 10 novel families, and six belonged to four novel orders. The most diverse phylum by far was *Actinobacteriota*, represented by 54 MAGs from four different classes (*Rubrobacteria*, *Thermoleophilia*, *Acidimicrobiia*, and *Actinobacteria*). When we clustered the MAGs based on a presence-absence matrix of encoded functions according to functional annotation via EggNOG orthologs (Fig. 3), most of the MAGs clustered according to their taxonomic affiliation, with some notable exceptions. Instead of clustering with the rest of *Actinobacteriota*, the *Rubrobacteria* and *Thermoleophilia* MAGs grouped with MAGs from the *Chloroflexota* phylum and a *Deinococcota* MAG (Fig. 3).

10.1128/mSystems.00786-20.5 FIG S1 Phylogenomic placement of MAGs representing previously unsequenced genera, families, and orders. Clade-specific trees were calculated based on concatenated amino acid alignment of 120 phylogenetic markers used in GTDB (8) using FastTree (starting tree, bio-neighbor-joining; model, Le-Gascuel). Alignment was filtered to contain sites conserved in 25% of sequences (4,911 positions for *Sphingomonadales*, 4,918 for *Acetobacterales*, 4,521 for *Gemmatimonadota*, 4,819 for *Cytophagales*, 4,118 positions for *Phycisphaerae*, 4,365 for *Chtoniobacterales*, 4,061 for *Chloroflexota*, 4,805 for *Armatimonadota*, 4,627 for *Thermosynechococcales*, 4,418 positions for *Rubrobacteria* and *Thermoleophilia*, 4,375 for *Acidimicriia*, 4,882 for *Mycobacteriales*, and 4,872 for *Propionibacterales*). Multifurcations have been introduced for branchings with less than 50% support. Branch support was calculated by 1,000 resamplings of site likelihood and the Shimodaira-Hasegawa test (FastTree default). Download FIG S1, TIF file, 1.5 MB. Copyright © 2021 Meier et al. 2021 Meier et al. This content is distributed under the terms of the Creative Commons Attribution 4.0 International license.

10.1128/mSystems.00786-20.10 TABLE S3 MAG metrics and classification. This large table provides sequence-related metrics of the metagenome-assembled genomes (MAGs), estimated values of their completeness and duplication, relative abundance of the MAGs in the individual metagenome samples, estimated indices of replication (iRep values), and taxonomic classification of the MAGs by GTDB-Tk and the criteria on which the classification was based (e.g., ANI to closest-related genome, if applicable). Download Table S3, XLSX file, 0.03 MB. Copyright © 2021 Meier et al. 2021 Meier et al. This content is distributed under the terms of the Creative Commons Attribution 4.0 International license.

### Potential for use of C sources and energy generation mechanisms.

**FIG 4:**
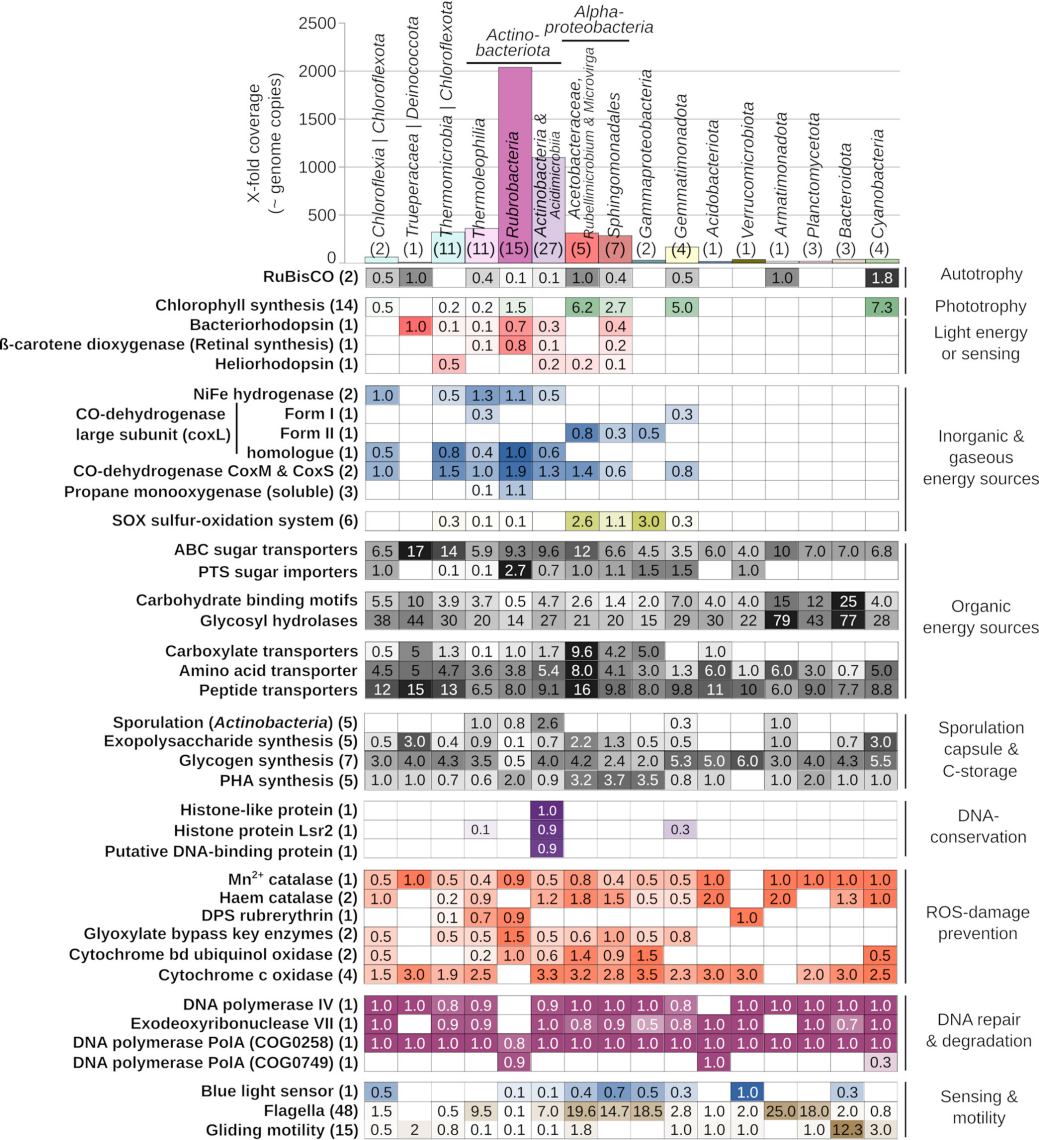
Selected metabolic traits encoded in the metagenome-assembled genomes. The genomes were grouped based on presence-absence of functions (see Fig. 2). The bar chart on top indicates the cumulative coverage of a given group of genomes in the whole data set. The number of genomes in a group is indicated in parentheses. On the left, the total number of enzyme subunits or pathway constituents is indicated in parentheses. No numbers are given for categories where a “completeness” criterion is not applicable, such as transporters of different specificity. The numbers in the table are the average number of different genes per MAG, like genes encoding (i) different subunits of an enzyme, (ii) different proteins of a pathway, or (iii) similar proteins of different specificity (e.g., different transporters, glycosyl hydrolases). Genes falling into shown categories can be found in Data Set S2.

In order to predict ecological roles of microbial community members, we first investigated the distribution of autotrophic, mixotrophic, and heterotrophic metabolic potentials encoded in the population genomes (Fig. 4; Fig. S2).

10.1128/mSystems.00786-20.6 FIG S2 Selected metabolic traits by MAG. The genomes were grouped based on a presence-absence matrix of EggNOG orthologs, which corresponds largely to taxonomic classification. For known pathways and enzyme subunits, numbers in parentheses indicate the number of different annotations in a given category, e.g., genes encoding different subunits of an enzyme or genes encoding different enzymes of a pathway. No numbers are given for categories where a “completeness” criterion is not applicable, such as transporters of different specificity. The numbers in the table indicate the number of genes per MAG. Several genes encoding the same function in a MAG, e.g., the same subunit of an enzyme, were counted only once. Genes falling into categories can be found in Data Set S2. Download FIG S2, TIF file, 1.3 MB. Copyright © 2021 Meier et al. 2021 Meier et al. This content is distributed under the terms of the Creative Commons Attribution 4.0 International license.

10.1128/mSystems.00786-20.2 DATA SET S2 Large spreadsheet listing the functional annotations, which were counted for the metabolisms depicted in Fig. 4 and Fig. S2. Download Data Set S2, XLSX file, 0.03 MB. Copyright © 2021 Meier et al. 2021 Meier et al. This content is distributed under the terms of the Creative Commons Attribution 4.0 International license.

Genes encoding the large subunit of ribulose-1,5-bisphosphate carboxylase (RuBisCO) indicative of CO_2_ fixation via the Calvin-Benson-Bassham (CBB) pathway were found in the four cyanobacterial MAGs, three *Alphaproteobacteria* MAGs (two *Acetobacteraceae* MAGs and the *Rubellimicrobium* MAG), two *Gemmatimonadota* MAGs, two *Thermoleophilia* MAGs, one *Rubrobacteria* MAG, one *Actinobacteria* MAG (genus *Pseudonocardia*), one *Acidimicrobiia* MAG, and one *Chloroflexia* MAG (Fig. 4; Fig. S2). Also, *Deinococcota* and *Armatimonadota* MAGs contained RuBisCO genes. No evidence for other CO_2_ fixation pathway*s* could be detected in our metagenomes (in MAGs or unassembled reads). Potential energy sources for CO_2_ fixation could be light or H_2_ oxidation.

In addition to the four *Cyanobacteria* MAGs, we found (bacterio)chlorophyll synthesis genes carried in three alphaproteobacterial MAGs belonging to the *Acetobacteraceae* family (two belonging to a novel genus and one belonging to *Belnapia*/*Craurococcus*) (Fig. 4; Fig. S2). Another two MAGs containing bacteriochlorophyll synthesis and RuBisCO genes were attributed to the *Gemmatimonadaceae*. Bacteriorhodopsins, which can be used to generate proton motive force or for light sensing, were found in 26 MAGs (Fig. S2). Genes for bacteriorhodopsin and ß-carotene di-oxygenase (a key enzyme in retinal synthesis) were present in most *Rubrobacteria* MAGs (in 11 and 12 MAGs, respectively), one *Thermoleophilia* MAG, four *Actinobacteria* MAGs (two *Mycobacteriales* MAGs, one *Actinomycetospora* MAG, and one *Quadrisphaera* MAG), and two *Alphaproteobacteria* MAGs (*Sphingomonadales*) (Fig. S2; Fig. 4). Seven various other MAGs encoded only the bacteriorhodopsin, and 12 MAGs encoded heliorhodopsins suggested to be involved in light sensing (37), among them five *Thermomicrobia* MAGs.

Apart from light, H_2_ oxidation seemed to be a common potential inorganic energy source. Of 27 detected hydrogenases, 23 were encoded by *Actinobacteriota*: *Rubrobacteria* (nine MAGs), *Thermoleophilia* (seven MAGs), *Acidiimicrobia* (two MAGs), and class *Actinobacteria* (five MAGs). Additionally, three hydrogenase-encoding genes were found in *Chloroflexota* MAGs. All hydrogenases belonged to the high-affinity group 1h [NiFe]-hydrogenase according to HydDB classification (38), indicative of the potential to oxidize atmospheric H_2_ (39). Genes encoding the form I CO dehydrogenase, experimentally shown to oxidize CO (40), were found in only four MAGs (Fig. S2). However, genes annotated as encoding CO dehydrogenase were widespread across *Chloroflexota*, *Actinobacteriota*, *Proteobacteria*, and *Gemmatimonadota* MAGs. Based on phylogenetic tree calculation (Fig. S3A), 5 of 59 annotated CO dehydrogenase genes were confirmed as encoding form II CO dehydrogenase. The function of the other CO dehydrogenase-like proteins could not be confidently determined, due to unresolved phylogeny of the functional domain. We have further identified genes annotated as encoding soluble methane monooxygenases (Fig. 4; Fig. S2). These genes (3 subunits) were found in seven *Rubrobacteria* MAGs, one *Actinobacteria* MAG, and one *Thermoleophilia* MAG. However, phylogenetic analysis suggests that they encode propane monooxygenases (Fig. S3B). Interestingly, two *Acetobacteraceae*, one *Methylobacteriaceae* (*Microvirga*, *Alphaproteobacteria*), and one *Comamonadaceae* (*Ramlibacter*, *Gammaproteobacteria*) MAG encoded the Sox multienzyme complex central to the oxidation of reduced sulfur species (Fig. 4; Fig. S2). While the bacteriochlorophyll-encoding *Acetobacteraceae* MAGs might potentially use sulfide as an electron donor in anoxygenic photosynthesis, the *Microvirga* and *Ramlibacter* MAGs did not carry genes indicative of phototrophy (Fig. S2).

10.1128/mSystems.00786-20.7 FIG S3 Phylogenetic placements of proteins annotated as carbon monoxide dehydrogenase, large subunit (A), and methane monooxygenase (B). Trees were calculated with FastTree based on an HMM alignment to the respective Pfam motif (PF02738 for CO dehydrogenase catalytic domain and PF02332 for putative methane monooxygenase). Multifurcations have been introduced for branchings with less than 50% support. Branch support was calculated by 1,000 resamplings of site likelihood and the Shimodaira-Hasegawa test (FastTree default). Numbers in parentheses indicate the number of sequences contained in the cluster (note that one MAG can have multiple CoxL-encoding genes). For the putative CO dehydrogenases (A), a first tree was calculated for all proteins included in the seed alignment of the Pfam model (excluding eukaryotes), together with known form I and form II CO dehydrogenases (9) and MAG-derived sequences. Alignment was filtered to contain sites conserved in 25% of sequences (428 positions). As the Pfam motif includes several enzymes with different functions, a subtree was calculated just for the CO dehydrogenase-containing part, using glyceraldehyde dehydrogenase as an outgroup. Alignment was filtered to contain sites conserved in 25% of sequences (502 positions). Download FIG S3, TIF file, 1.2 MB. Copyright © 2021 Meier et al. 2021 Meier et al. This content is distributed under the terms of the Creative Commons Attribution 4.0 International license.

We have not found any indications for anaerobic respiration in any of the analyzed MAGs. Therefore, oxygen seems to be the only electron acceptor for respiratory processes. An overwhelming majority of the MAGs encoded heme-copper-based cytochrome *c* terminal oxidases, with a few MAGs additionally encoding cytochrome *bd* ubiquinol oxidases. Interestingly, *Rubrobacteria* MAGs did not encode any components of the cytochrome *c* oxidase, only the cytochrome *bd* ubiquinol oxidase. All genomes had genes encoding enzymes involved in at least one branch of mixed-acid fermentation. However, most enzymes involved in fermentation are bidirectional and also part of other metabolic pathways. Therefore, no prediction can be made about the ability of the populations to perform fermentation.

When looking for organic energy and C sources, we focused on transporters, as they determine the binding and uptake of organic substrates from the environment. MAGs of all groups encoded ATP-dependent sugar transporters of different specificities (Fig. 4; Fig. S2). Compared to other groups, *Rubrobacteria* MAGs had a high number of different phosphotransferase systems (on average, 2.7/MAG, up to a maximum of 8). The most common substrates targeted by the phosphotransferase systems were fructose, mannitol, sorbitol, and *N-acetyl-*d-glucosamine, according to RAST annotation. The mannitol-specific phosphotransferase system was found encoded in seven of the *Rubrobacteria* MAGs and only in five other MAGs (from the class *Actinobacteria*). At the same time, *Rubrobacteria* MAGs encoded strikingly few proteins with carbohydrate-binding motifs (on average, 0.5/MAG) (Fig. 4; Fig. S2) compared to other MAGs. High numbers of different carbohydrate-binding motifs and glycosyl hydrolases indicating an ability to utilize complex polymeric C substrates were detected in *Bacteroidota*, *Armatimonadota*, *Planctomycetota*, and *Deinococcota* MAGs (Fig. 4; Fig. S2). Amino acid and peptide transporters were relatively evenly distributed between different MAGs (Fig. 4; Fig. S2).

### Genes involved in microbial dormancy and persistence.

We investigated the MAGs for the potential of actinobacterial sporulation by searching for genes essential for this sporulation type, such as those encoding sporulation-specific cell division activator SsgA and transcriptional regulators WhiA, WhiB, and WhiD (41, 42), as many other involved genes also can have roles beyond sporulation. As expected, the highest frequencies of these genes were detected in actinobacterial MAGs, more specifically in the classes *Acidimicrobiia* and *Actinobacteria*. In the MAGs belonging to these classes, we also found genes encoding histone-like proteins, possibly involved in DNA compaction and protection in a resting state (43, 44) (Fig. 4; Fig. S2). In contrast, MAGs belonging to the actinobacterial classes *Thermoleophilia* and *Rubrobacteria* were mostly lacking sporulation-related genes (Fig. 4; Fig. S2). Homologs of genes involved in the sporulation of *Firmicutes* were found in several MAGs (data not shown). However, their functions were not restricted to sporulation, their sets were far from complete, with essential key genes missing, and they seemed to be randomly distributed across MAGs. Therefore, we cannot conclude that the potential for *Firmicutes*-like sporulation was present in any of the MAGs. We further searched the MAGs for the potential to protect the cells with polysaccharide sheaths or capsules. Genes for exopolysaccharide production were present in several MAGs from various groups, most frequently in *Deinococcota*, *Acetobacteraceae*, and *Cyanobacteria* (Fig. 4; Fig. S2), but were lacking in all *Rubrobacteria* MAGs. Polymeric C storage compounds like glycogen or polyhydroxyalkanoates (PHA) are known to play an important role in successful formation of and resuscitation from resting stages (45–47). *Rubrobacteria* MAGs carried only genes for PHA synthesis, whereas most other MAGs carried genes for glycogen synthesis and modification. Among *Alphaproteobacteria*, *Sphingomonadales* MAGs were largely lacking glycogen synthesis-related genes.

Protein and DNA damage induced by reactive oxygen species (ROS) is considered the main mortality cause during desiccation (48, 49), and as such, we compared the genomic potentials for damage mitigation and repair. While many MAGs encoded heme-based catalases and peroxidases (KatE and KatG), *Deinococcota*, *Rubrobacteria*, and *Planctomycetota* encoded only manganese-containing catalases (Fig. 4; Fig. S2). Almost all *Rubrobacteria* and *Thermoleophilia* MAGs as well as one *Thermomicrobia* and the *Verrucomicrobiota* MAG additionally encoded a “DNA protection during starvation” (DPS) rubrerythrin-like protein, which contains a DNA-binding domain and has a peroxidase function (50). The exclusive use of cytochrome *bd* terminal oxidase, which has been shown to have significant catalase activity (51), might represent an additional ROS-reducing feature of the *Rubrobacteria* MAGs (Fig. 4; Fig. S2). The two key enzymes of the glyoxylate bypass of the tricarboxylic acid (TCA) cycle allowing the cells to utilize C_2_ compounds (here, malate synthase and isocitrate lyase) were encoded in most *Rubrobacteria* (11 MAGs encoded both enzymes, one MAG encoded only one) and *Sphingomonadales* MAGs (all encoded at least one enzyme, three MAGs encoded both enzymes) (Fig. 4; Fig. S2). Glyoxylate bypass was suggested to play a role in oxidative stress resistance, as its upregulation has been observed in oxygen stress conditions (52). Most of the MAGs had a similar potential for DNA repair, whereas *Rubrobacteria* and *Acidobacteriota* MAGs were missing genes for polymerase IV and exodeoxyribonuclease VII (Fig. 4; Fig. S2), both involved in “reckless degradation” of damaged DNA (53, 54). Furthermore, *Rubrobacteria* and *Acidobacteriota* encoded an additional DNA polymerase PolA protein that differs from the common PolA. PolA plays a crucial role in double-strand break repair (55).

### Genomic potential for light sensing and motility.

We found genes encoding blue light sensor proteins in 19 MAGs, which were most frequent among the *Proteobacteria* (9 out of 14 MAGs). Twenty-three MAGs carried five or more genes for flagellum assembly (22 of them more than 10 genes), indicating the potential to move in liquid medium or attach to surfaces (Fig. S2; Fig. 4). Of these MAGs, six encoded both blue light sensor and flagellar proteins (one *Actinobacteria*, three *Sphingomonadales*, and two *Acetobacteraceae* MAGs). Three further MAGs (one *Quadrisphaera* and two *Sphingomonadales*) encoded flagellar proteins and a rhodopsin, which can be used for sensing light (56). The three putatively phototrophic *Acetobacteraceae* MAGs contained gliding motility genes (Fig. S2; Fig. 4), some of which were also carried by all cyanobacterial MAGs and two *Bacteroidota* MAGs. Notably, *Rubrobacteria* MAGs contained hardly any motility-related or blue light sensing genes (Fig. S2; Fig. 4).

### Genome comparison of *Rubrobacteria* from BSC and aquatic habitats.

**FIG 5:**
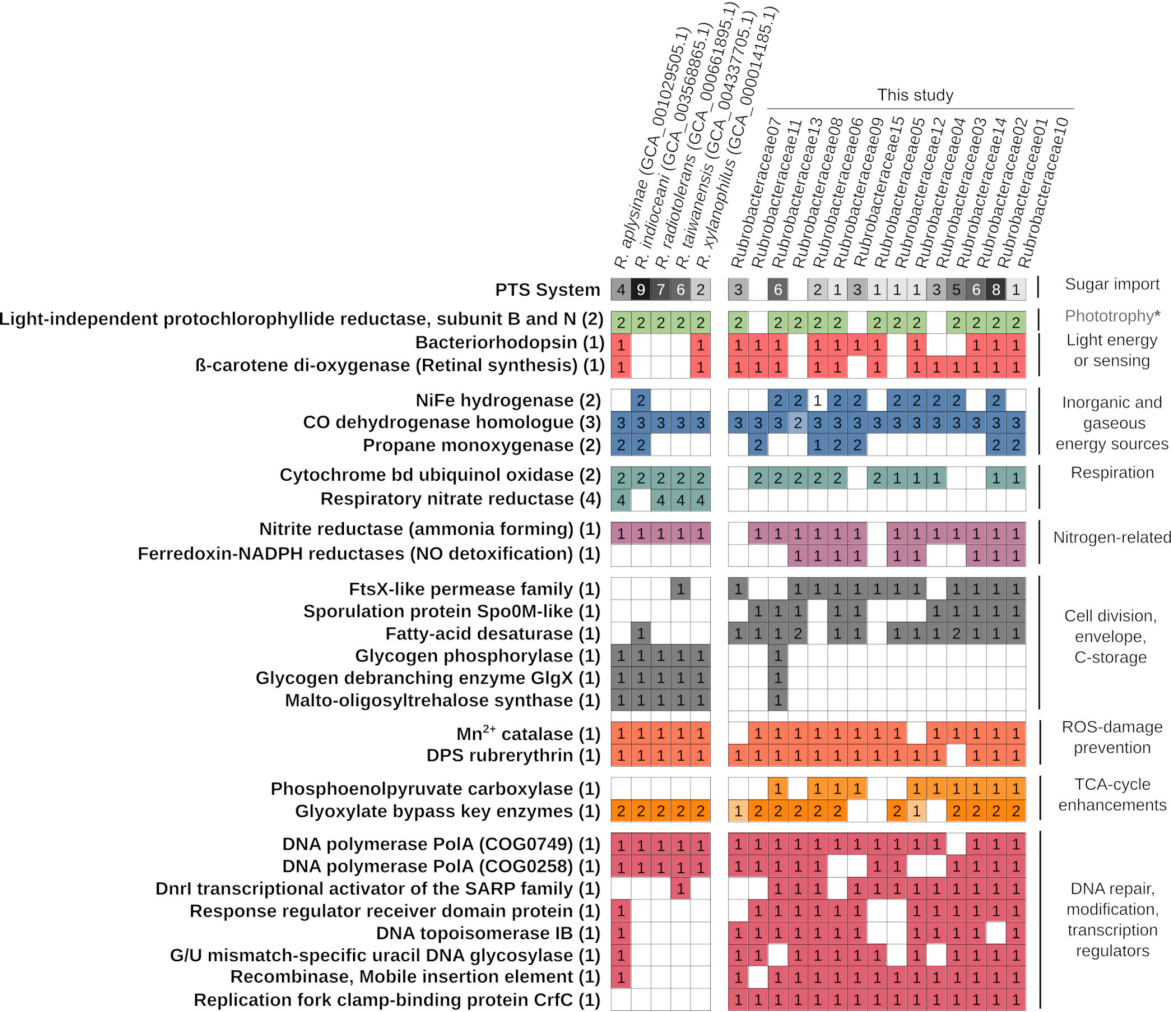
Comparison of selected metabolic traits between *Rubrobacteraceae* MAGs from this study and sequenced genomes of *Rubrobacteraceae* isolates. The analysis is based on comparison of EggNOG orthologous groups detected in the genomes. In cases where the EggNOG/COG functional annotation was too general, annotations were completed based on specific RAST, Pfam, and UniProt search results for the given genes. The asterisk indicates that the genes encoding light-independent protochlorophyllide reductase subunits B and N do not represent a potential for phototrophy, as they are the only bacteriochlorophyll synthesis genes found in *Rubrobacter* genomes. The isolate genomes stem from aquatic environments, such as a Mediterranean sponge (Rubrobacter aplysineae) (57), Indian Ocean sediment (Rubrobacter indicoceanii) (58), mud and thermo-mineral water of a radioactive spring (Rubrobacter radiotolerans) (59), hot spring water, and thermally heated mud and soil (Rubrobacter taiwanensis) (60), and biofilm of a thermally polluted runoff of a carpet factory (Rubrobacter xylanophilus) (61).

Due to the high abundance of *Rubrobacteria* MAGs (all closely related to genus *Rubrobacter*) in the investigated BSC metagenomes, we compared the functional potential encoded in these MAGs to that of previously sequenced genomes of *Rubrobacter* isolates (Fig. 5). We aimed to (i) investigate whether the special features we found in our BSC MAGs are common to all *Rubrobacteraceae* genomes and (ii) identify desert soil-specific adaptations, since all the previously sequenced *Rubrobacter* strains originated from aquatic environments (Fig. 5) (57–61).

Among the common features we identified were the following: the presence of two chlorophyll synthesis-related proteins (light-independent protochlorophyllide reductase subunits B and N), the presence of a CO dehydrogenase-like protein, the absence of terminal cytochrome *c* oxidase and the use of the cytochrome *bd* ubiquinol oxidase instead, reliance on manganese catalase only, and the presence of the rubrerythrin-like DPS protein, the enzymes of the glyoxylate bypass, and the two copies of the DNA polymerase PolA essential for double-strand break repair (Fig. 5).

Differences between BSC MAGs and aquatic isolates were detected in the distribution of bacteriorhodopsins, which seemed to be more widespread in the BSC MAGs (11 in 15 MAGs compared to two in five isolate genomes). Likewise, a group 1h [NiFe]-hydrogenase was found encoded in only one isolate genome but in the majority of the BSC MAGs (Fig. 5). However, the most striking difference was the complete absence of respiratory nitrate reductase genes in the BSC MAGs, whereas these were present in every isolate genome. Further significant differences were the cell envelope-related proteins FstX-like permease, a Spo0M (a *Firmicutes* sporulation protein) homolog, and a fatty acid desaturase, which were found encoded only in the BSC MAGs. Unlike isolate genomes, the BSC MAGs were missing genes related to synthesis and modifications of glycogen and carried genes encoding phosphoenolpyruvate carboxylase. This anapleurotic enzyme replenishes oxaloacetate in the TCA cycle, thus accelerating utilization of organic C. Finally, the BSC MAGs encoded several proteins related to transcription regulation, DNA modification, and mobile elements, which were absent from almost all aquatic isolate genomes (Fig. 5).

### Indices of replication.

We analyzed the contig coverage to identify potentially actively dividing populations using iRep (62). For any MAG that passed the criteria for iRep calculation in any sample, the indices suggested that at least 40% of the cells (iRep > 1.4) were dividing (Table S3). For many MAGs, replication with multiple replication forks was indicated (Table S3), and two *Rubrobacteraceae* and one *Solirubrobacteraceae* MAG reached values over 3. There was no significant difference between dry and hydrated samples.

## DISCUSSION

To the best of our knowledge, this is the first comprehensive, large-scale population-resolved metagenomics study linking metabolic potential to microbial populations in arid BSCs. Genomic bins were previously generated for support in a metabolomics-centered study (15) and in a study of the same data set addressing bacteriophages blooms (63), where the functional potential of the overall community based on generated MAGs was beyond the focus of the studies. Here, we analyzed the metabolic potential of MAGs representing the most abundant BSC microbial taxa (Fig. 2), including entirely uncultured and previously unsequenced orders, families, and genera (see Table S3 and Fig. S1 in the supplemental material). Thereby, we were able to not only identify potential physiologies regarding energy generation and dormancy mechanisms but to link these with the respective microbial populations. This study establishes a foundation that allows the generation of hypotheses about the activities and interactions of BSC community members and their future testing.

### Widespread distribution of CO_2_ fixation genes in BSC populations—beyond the cyanobacterial realm.

We detected the potential for CO_2_ fixation via the Calvin-Benson-Bassham (CBB) cycle in several populations from six different phyla and a widespread potential for utilization of inorganic energy sources such as light and atmospheric gases among the MAGs (Fig. 4; Fig. S2). This widespread distribution suggests that trophic relations in the investigated BSC microbial community might extend beyond the dependency between cyanobacteria as primary producers and various heterotrophs consuming their products (14, 15). While an early metagenomic study of BSCs found cyanobacteria as the only CO_2_-fixing organisms (25), other culture- and marker gene-based studies of crusts and arid soils also detected anoxygenic phototrophic *Alphaproteobacteria* (23, 64). In addition to *Alphaproteobacteria*, we found potential for anoxygenic photosynthesis among *Gemmatimonadota*, a phylum that was only recently discovered to contain phototropic species (65). Further, we found populations with chemolithoautotrophic metabolic potential involving H_2_ oxidation among, e.g., *Thermoleophilia*, *Acidimicrobiia*, and other *Actinobacteriota.* The potential for chemolithoautotrophic CO_2_ fixation (e.g., by the aforementioned *Thermoleophilia*, *Acidimicrobiia*, and other *Actinobacteriota*) could make these organisms independent from cyanobacterial C input; however, it is unclear whether the energy from atmospheric gas oxidation is sufficient to produce surplus organic C that could support obligate heterotrophic community members.

Although previous studies reported indications of CO_2_ fixation pathways other than the CBB cycle in desert soil communities (28, 30, 66), we did not find any genomic potential for additional pathways, such as the reverse TCA cycle or the reductive acetyl coenzyme A (acetyl-CoA) pathway, encoded in our MAGs. Yet, there are likely low-abundance populations that use other pathways to fix CO_2_, e.g., sulfate-reducing Deltaproteobacteria and nitrifying or methanogenic *Archaea.* These groups were previously reported in BSCs based on process rate measurements and marker gene-based studies (19, 22, 67) and were detected as very low-abundance OTUs in the 16S rRNA amplicon data (Data Set S1).

### Potential for atmospheric H_2_ oxidation is more widespread than for atmospheric CO oxidation.

Recent studies have shown that the oxidation of atmospheric trace gases can be an alternative energy source for soil microorganisms (39, 68, 69), and it has been hypothesized that it might be widely used to generate maintenance energy during dormancy (11). We detected MAGs with the potential for H_2_ oxidation in the phyla *Actinobacteriota* and *Chloroflexota*, consistent with previous investigations (70), and H_2_ oxidation was recently detected in BSCs from the same sampling site (133). As mentioned above, some actinobacterial MAGs also harbored the genes for CO_2_ fixation, suggesting that H_2_ oxidation may be a means to generate energy for CO_2_ fixation, as suggested by Ji et al. (12). However, we also detected the potential for H_2_ oxidation in populations without the potential to fix CO_2_, indicating its utilization solely as a supportive energy source. The genetic potential for CO oxidation (via form I CO dehydrogenase) was far less common than previously hypothesized (11, 68), as form I CO dehydrogenase genes were detected in only four MAGs within the phyla *Actinobacteriota* and *Gemmatimonadota*. Instead, genes homologous to the CO dehydrogenase genes were found in many populations within the *Actinobacteriota* and *Gemmatimonadota*, as well as genes encoding form II CO dehydrogenases in MAGs of *Alphaproteobacteria* and *Gemmatimonadota*. The form II CO dehydrogenase is homologous to form I and seems to differ in its affinity for CO: it has been suggested that CO oxidation might not be its primary function (40). As such, these putative CO dehydrogenase-oxidizing enzymes require follow-up investigations to ascertain if they are functioning as a true CO dehydrogenase. Taken together, it appears that the genomic potential for atmospheric H_2_ scavenging is more ubiquitous than for CO scavenging in this arid BSC.

### Limited genomic potential for polymeric C degradation but widespread potential for use of small organic substrates in BSC genomes.

We detected the potential for degrading polymeric polysaccharides in MAGs from the phyla *Bacteroidota*, *Armatimonadota*, and *Planctomycetota* (Fig. 4; Fig. S2), which is consistent with previous investigations of representatives from these phyla in soils and other environments (71–73). Populations encoding this physiological potential were of relatively low abundance in the BSC (Fig. 4; Fig. S2), suggesting that this physiological potential is limited in the BSC. In contrast, the genes for uptake of small organic substrates, such as oligopeptides, amino acids, and sugars, appear to be widespread (98% of MAGs) in the community (Fig. 4; Fig. S2). It is noteworthy that the polysaccharide-specialized MAGs carried no genes for energy generation from inorganic energy sources (such as H_2_ or CO). Polysaccharides excreted by cyanobacteria and other microorganisms are one of the largest C pools in BSCs (13). The ability to utilize such complex substrates might provide the cell with ample organic C and energy during active phases, enabling them to build up storage compounds and alleviate the need for additional energy sources.

### Most BSC populations use non-sporulation persistence strategies.

The majority of the genomes (based on MAG coverage) across many taxa in our data set (e.g., *Thermoleophilia*, all *Chloroflexota*, *Gemmatimonadota*) did not carry genes indicative of a classical resting-stage formation. Such genomic potential was encoded only in a few MAGs belonging to the classes *Acidimicrobiia* and *Actinobacteria* (phylum *Actinobacteriota*) that contain known spore-forming species (41, 42). *Firmicutes*, another prominent spore-forming phylum that was reported to bloom in BSCs upon hydration (63, 74), was present only at very low relative abundances (based on 16S rRNA gene amplicon and unassembled metagenome data) (Fig. 2), which likely precluded the generation of MAGs. The lack of a *Firmicutes* bloom might be explained by different rehydration conditions, e.g., only to ca. 75% water-holding capacity in this study (e.g., 350 μl added per 1 g crust), while crusts in which *Firmicutes* blooms were reported were inundated with water (1 ml added to 0.5 g crust) (15, 63). However, it is possible that genes involved in resting-stage formation in some of our MAGs were not homologous to sequences in public databases, and as such we were unable to detect them with confidence. Nevertheless, we found indications of desiccation-adapted cell envelopes in other MAGs, such as polysaccharide sheaths in *Cyanobacteria* (75), which was supported by detected exopolysaccharide synthesis genes (Fig. 4). In addition, exopolysaccharide synthesis genes found in *Acetobacteraceae* MAGs (Fig. 4) suggest the ability to form alginate-reinforced cysts, as known from some *Alphaproteobacteria* species (47). The missing sporulation genes in *Thermoleophilia* and *Rubrobacteria*, which are part of *Actinobacteriota*, suggest that they do not undergo morphological transformations in preparation for desiccation, as supported by *Rubrobacter* cultures (61, 76, 77). Accordingly, *Rubrobacteria* MAGs also did not contain the genes for glycogen storage buildup (Fig. 4). However, we found a high frequency of bacteriorhodopsin- and hydrogenase-encoding genes in the *Rubrobacteria* MAGs, indicating that they could use light and atmospheric H_2_ as alternative energy sources during starvation. Hydrogenase-encoding genes were more widespread among *Rubrobacteria* MAGs obtained in this study than in previously sequenced *Rubrobacteria* genomes stemming from aquatic environments (Fig. 5). This adaptation seems plausible, since the accessibility of atmospheric H_2_ is enhanced by increased gas exchange in dry, porous desert soil.

### *Rubrobacteria* genomes in BSC are particularly equipped for desiccation survival.

**FIG 6:**
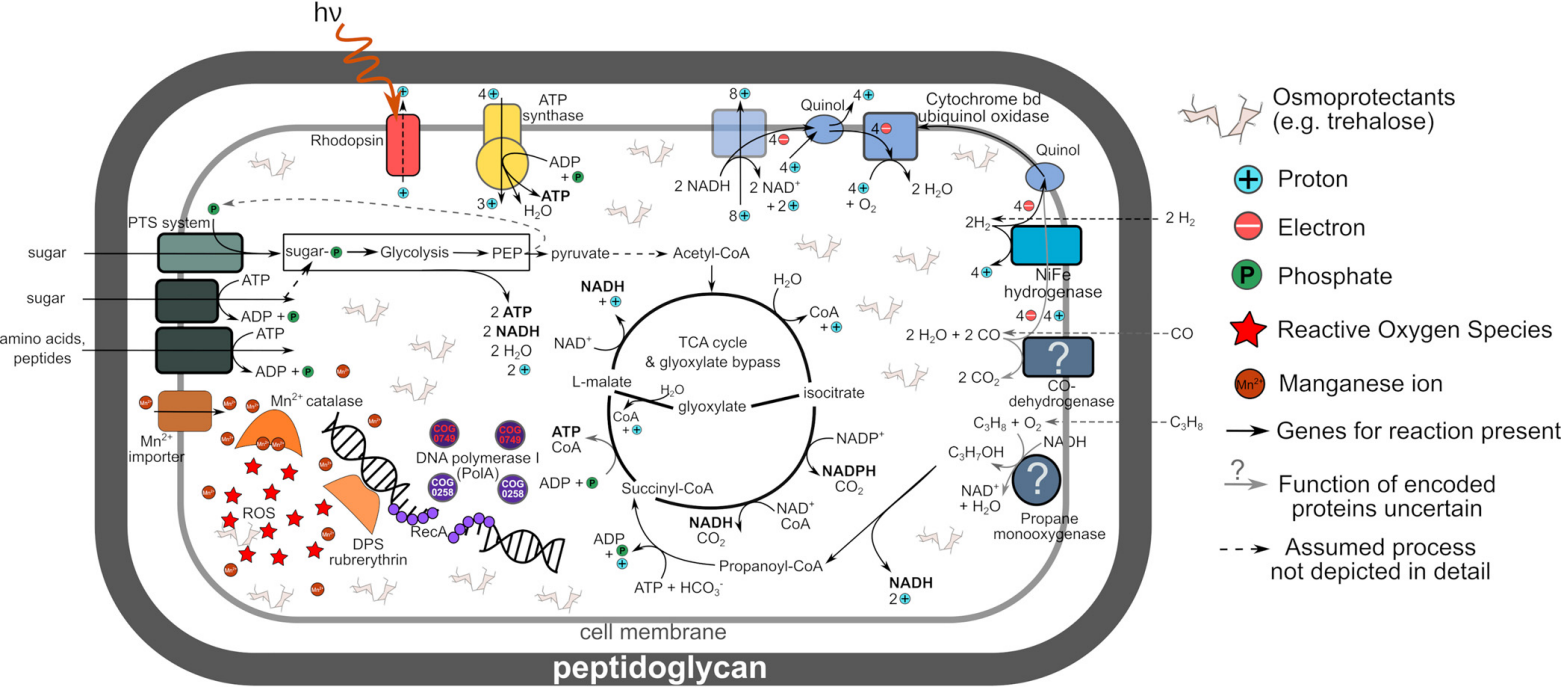
Exemplary metabolic sketch of BSC *Rubrobacteraceae* species based on the most abundant MAG. The sketch illustrates basic energy generation and C acquisition mechanisms, as well as some stress resistance and survival mechanisms. *Rubrobacteraceae* encode genetic potential for a mixotrophic lifestyle with rhodopsin and a high-affinity hydrogenase to generate proton motive force. The exact functions of CO dehydrogenase and propane monooxygenase homologs are yet to be determined. The C metabolism is optimized for efficient C utilization by using phosphotransferase sugar import systems (PTS) and glyoxylate bypass of the TCA cycle. It is important to note the absence of terminal cytochrome *c* oxidase in the respiratory chain and exclusive reliance on the manganese-based catalase for combating reactive oxygen species (ROS). DNA-binding rubrerythrin might also contribute to preventing ROS damage to DNA. Also note the presence of an additional homolog of PolA polymerase (COG0749), key in double-stranded DNA break repair. For pathway evaluation, RAST annotations were analyzed with Pathway Tools v.24 (131), and missing enzymes, usually due to the absence of an EC number in the annotation or the use of an uncommon synonymous enzyme name, were searched manually in RAST and other annotations of the MAGs. The depicted pathways are simplified versions of pathway depictions in the MetaCyc database (132) used by Pathway Tools software.

Hereafter, we discuss the genomic potential of the most abundant group of MAGs in the data set, *Rubrobacteraceae* (Fig. 6), from the perspective of desiccation survival. While light and H_2_ oxidation may provide additional energy (Fig. 6) as water and organic C availability decreases, it is unclear if the low soil water content during the dry period allows any ongoing enzyme activity. While pockets of increased humidity have been proposed to exist in dry soil (5, 66), a study applying *in situ* X-ray tomography to BSCs from the Moab Desert suggested the absence of such pockets after complete desiccation (78). However, even the near complete absence of water does not have to be fatal for a microbial cell. The damage by low water content can be mitigated by small organic molecules, like trehalose or other sugars (Fig. 6), that replace water in the cell, maintaining hydrogen bonds and preserving the structure of proteins (79), a principle used for storage of freeze-dried microbial culture stocks. Rubrobacter xylanophilus has been shown to accumulate and maintain unusually high intracellular concentrations of the osmoprotectants trehalose and mannosylglycerate by default under any growth condition (80). *Rubrobacteria* cells could thus be permanently prepared for desiccation by maintaining these high concentrations of osmolytes (Fig. 6) and therefore do not need to transform into a dedicated resting stage. In our study, we found a large variety of sugar-importing phosphotransferase systems encoded in *Rubrobacteria* MAGs that could be beneficial for such a strategy. Extensive studies of extreme radiation and desiccation tolerance of Deinococcus radiodurans have shown that its cells reassemble the fragmented genome from multiple mutual-correcting copies upon rehydration (55, 81), while ROS-induced damage in the desiccated state is reduced by accumulating manganese ions and ROS-scavenging molecules (48). The *Rubrobacteria* MAGs showed several indications of a similar strategy. Most of their characteristic genomic features were related to minimizing ROS accumulation by using antioxidant proteins like manganese catalase, DPS rubrerythrin (Fig. 6), and a different terminal oxidase (Fig. 6) with an additional catalase function (51). BSC *Rubrobacteria* MAGs encoded an additional version of DNA double-strand break repair polymerase PolA (55). The lack of “reckless” DNA degradation enzymes (53) might be a way to exclude even the possibility of unnecessary degradation of damaged DNA that could still be repaired.

### Challenges in using the index of replication as a measure of activity in BSC microorganisms.

The index of replication (iRep) was suggested as a way to estimate the replication status of microbial genomes based on their coverage in metagenomes. It is based on the assumption that regions of the genome closer to the origin of replication should have a higher coverage when a population containing actively dividing cells is sequenced (62). Irrespective of whether BSCs were dry or rehydrated, all MAGs that passed the threshold for iRep calculation were indicated as actively dividing (Table S3). Similar values have been reported for the dry Atacama soil and interpreted as indicators of active cell division (3). However, in environments where the activity of cells is restricted by water availability, the principle of the iRep approach might be impaired. Possibly, cellular processes such as DNA replication pause when the cell dries out and resume upon a water pulse. In this scenario, high iRep values might reflect a state of paused DNA replication. Additionally, high iRep values may also be caused by high strain diversity in the samples, as genome regions conserved across all strains would have higher coverage than more divergent regions (62).

### Population-resolved metagenomics as a stepping stone in BSC microbial ecology.

The high degree of genomic novelty revealed in this study of BSCs from the Negev Desert illustrates the importance of metagenomic sequencing of terrestrial environments. In contrast to well-catalogued human microbiomes, e.g., from the oral cavity (82) and gut (83), where DNA or RNA sequence reads can readily be mapped to existing genomes, reference genomes are still largely missing for the highly diverse soil communities (84). This missing information also hinders detailed studies of, e.g., microbial activity by transcriptomics. Additionally, assuming the function of an organism based on the closest cultured or sequenced relatives can be misleading, as illustrated by a couple of examples in our data set. First, based on taxonomy, many of the detected microorganisms could potentially be diazotrophs, such as *Frankiales* (*Actinobacteria*) (85), *Microvirga* (86), and *Acetobacteraceae* (*Alphaproteobacteria*) (87). But none of the MAGs assigned to these groups contained genes involved in N_2_ fixation, which does not exclude the possibility that other, lower-abundance MAGs in these groups might carry this genomic potential. However, the absence of such genes not only among the most abundant microbial populations represented by MAGs but also in the unassembled reads illustrates the low relative abundances of these important community members in the *Microcoleus*-dominated crusts. Similar observations on missing sequence coverage of diazotrophs have been made in metagenome libraries of ocean waters with known N_2_ fixation activity (88, 89). Another example is the process of denitrification that has been detected in BSCs, ranging from very low rates in North American BSCs (90) to high rates in BSCs from Oman (91). The ability to respire nitrate was previously found in all sequenced *Rubrobacteria* genomes stemming from aquatic environments (60, 61, 77, 92), and without genome information of BSC *Rubrobacteria*, one might have assumed this abundant group encoded this physiological potential. However, genes involved in denitrification were completely absent from *Rubrobacteria* MAGs and our complete data set, despite *Rubrobacteria* genomes being by far the most abundant ones.

The above-mentioned discrepancies show that population-resolved metagenomic analysis was essential to gain insights into the distribution of potential lifestyles and survival strategies within the microbial populations constituting the BSC communities.

### Metagenomics as a tool for hypothesis generation.

Our extensive analysis of population-resolved metagenomic data from BSCs in the Negev Desert indicates that a spectrum of different desiccation survival potentials is simultaneously present among the BSC genomes. Components of this spectrum can exist in pure form or can be combined to various degrees in one organism. On the one hand, there are genomes with strictly heterotrophic genomic potential, e.g., those of *Bacteroidota* and *Planctomycetota*, and genomes encoding resting-stage formation, e.g., in some *Actinobacteria* and *Alphaproteobacteria*. On the other hand, there are genomes with the potential to use continuously available inorganic energy sources and/or to preserve the cell components with a matrix of small organic molecules that would accumulate upon evaporation of water. Such strategies could be used for survival instead of a morphological transformation into a dedicated resting stage. The majority of the microbial populations encode metabolic potential for a combination of both strategies. Potential energy sources of most members of the BSC microbial community include both inorganic and organic energy sources. The potential to use inorganic energy sources and the potential to fix CO_2_ found in several populations might at least partially decouple these microorganisms from the primary production of cyanobacteria. How these different metabolic potentials are implemented and what implications they have for resuscitation of individual populations and for the long-term maintenance of the microbial community structure can now be tested by activity-based studies targeting specific populations.

## MATERIALS AND METHODS

### Sample collection.

Biological soil crust samples were collected in June 2017 at the long-term ecological research site (LTER) Avdat (30°36′33″N, 34°44′48″E), Negev Desert, Israel. Descriptions of basic soil properties and microbial community composition can be found, e.g., in references 93 to 96. With approximately 100 mm of yearly precipitation, the crusts at the site are light in color and dominated by cyanobacteria and contain little to no lichens and mosses, which, having large genomes, might significantly reduce the coverage of bacterial and archaeal genomes.

Soil crust samples were collected from 0.5- by 0.5-m patches distributed across the LTER with a distance of over 20 m between patches (Fig. 1). The sampling positions were chosen in shrub-free areas with a homogeneous surface appearance of the crust. We defined the cohesive crust layer of ca. 5 mm thickness on top of the soil as the crust. The pieces were carefully collected by hand and placed into petri dishes, which were immediately sealed with Parafilm. The crust samples were shipped and stored at room temperature in the dark until DNA extraction (4 weeks in total). Before crust pieces were weighed for DNA extraction, loose soil, if present, was removed from below the crust with a spatula. The thickness of extracted pieces was ca. 3 to 5 mm.

### DNA extraction and sequencing.

DNA was extracted from ca. 500 mg of soil crust with a harsh phenol-chloroform-based protocol, including three bead-beating and extraction steps and CTAB (cetyltrimethylammonium bromide)-based removal of organic polymeric substances (97).

Extracted DNA was first used for 16S rRNA gene fragment amplification and Illumina MiSeq sequencing as described by Herbold et al. (98).

### 16S rRNA gene amplicon analysis.

The sequences were error-corrected using the Bayes-Hammer module (99) of SPAdes assembler v.3.11 (100), paired reads were merged using BBmerge v.37.61 (101) with the “strict” setting and a minimum overlap of 50 bp after clipping 3′ ends with quality scores below 20, and amplicon sequence variants (ASVs) were determined using DADA2 (102) with standard settings. The ASVs were further grouped into percentage identity-independent “operational taxonomic units” with Swarm2 (33) in fastidious mode with the limit of a large swarm for grafting set at 20. Taxonomy was assigned to OTU centroids by the last common ancestor (LCA) algorithm using rRNA secondary structure-aware SINA aligner v.1.2.11 (103) and the SILVA SSU132 database (104). OTUs classified as mitochondria or chloroplasts (21 in total) were removed, since the primers are not designed and optimized to accurately capture eukaryotic diversity. Clustering and plotting of data were performed in R using the packages vegan (105) and ggplot2 (106).

### Choosing samples for metagenomic sequencing.

Assignment of metagenomic contigs to genomes (binning) is to a large extent based on genome-specific patterns of varying relative abundance across samples. Thus, based on the community composition determined by 16S rRNA amplicon sequencing, we chose three DNA extracts for metagenome sequencing, with the aim to cover most of the diversity and to have different relative abundances of taxa in the samples to enable differential-coverage genome binning. Additionally, we wanted to evaluate the practicability of using read coverage of metagenome bins to estimate the proportion of dividing cells by calculating the indices of replication. Since we expected cell division to increase in the hydrated state (which should be detectable in a change in genome replication indices), we rehydrated two additional crust pieces from plots where we picked dry pieces for metagenomic sequencing. Water was added to crust pieces to reach up to 26% water content (v/m_wet_, 75% of water-holding capacity of these crusts), and crusts were then incubated for 24 h in a sealed petri dish under 12 h of light (27°C) and 10 h of darkness (19°C) with 1-h transition periods in between in a climate-controlled chamber (Aralab, Rio de Mouro, Portugal). DNA was extracted the same way as done from the dry crusts. DNA was sequenced on an Illumina HiSeq 2500 instrument at the Vienna Biocenter Core Facility in the 2 × 150 bp read mode.

### Microbial community composition and diversity estimation based on metagenomic reads.

To estimate the diversity and coverage of our metagenomic libraries, we analyzed the reads with Nonpareil v.3.303 (34) with default settings using the k-mer-based overlap search and fastq input. To estimate the taxonomic diversity in the libraries, reads were mapped to the SILVA SSU132 database using PhyloFlash v.3.0 (107). Taxa were considered detected when three or more reads were collected. We consider this method more precise than to estimate taxa based on protein blast hits to the NCBI nr database, as the SILVA database contains many more taxa than the genome-based NCBI nr database does. The rRNA gene is a well-established phylogenetic marker, whereas not all proteins encoded on the contigs can serve as such and can create noise when used for classification of raw reads or contigs.

### Metagenome assembly and binning.

A detailed description of the logic behind the assembly and binning process is provided in Text S1 in the supplemental material and on https://github.com/meierdv/avdat_metagenome. Bash and R scripts with the commands used can be found on github at https://github.com/meierdv/avdat_metagenome.

Sequence reads were trimmed using BBduk v.37.61 (101), error-corrected using the Bayes-Hammer module (99) of SPAdes assembler v.3.11 (100), normalized to a target k-mer depth of 33× with BBnorm, and coassembled using MEGAHIT v.1.1.2 (108) with k-mers ranging from 21 to 137 in steps of 10. Contigs below 1,000 bp were removed from the final assembly. Error-corrected, not normalized reads were mapped to the contigs with BBmap v.37.61 (101) using an identity cutoff of 95% to assess the true relative abundance of contigs in each data set. Contigs were taxonomically classified using diamond blastp v.0.9.10 (109) on the translated open reading frames (ORFs predicted by Prodigal v.2.6.2) (110) against the NCBI nr protein database. The contigs were binned with MetaBAT v.2.12.1 (111), MaxBin v.2.2.4 (112), CONCOCT v.0.4.1 (113), and Metawatt v.3.5.3 (114). The results were summarized using DAStool v.1.1.0 (115), and the bins were analyzed with CheckM v.1.0.7 (116). Bins were grouped based on their placement in the CheckM reference genome tree and used as references for read mapping in order to perform reassemblies using less-complex clade-specific read sets and the more computationally intensive metaSPAdes v.3.11 assembler (117), with k-mers ranging from 21 to 127 in steps of 10.

The idea of improving assembly and bins by targeted reassembly is based on the logic described by Albertsen et al. (118). Generated reassemblies of the different taxonomic groups were binned in the same way as the coassembly. The binning was inspected and refined manually in Anvi'o v.5.2 (114). Mainly, contigs clearly clustering apart from the bin, based on the combination of tetranucleotide frequencies and differential coverage patterns, were removed from the bins. New bins were compared to the corresponding initial bins with dRep v.1.4.3 (119), and the ones with the better metrics (bigger size, higher completeness, lower contamination) were kept. Bins were filtered based on completeness (>50%) and contamination (<10%) estimated with CheckM (116), resulting in a final set of 96 metagenome-assembled genomes (MAGs).

### MAG annotation and taxonomic classification.

First, the MAGs were annotated using the RAST-Tk pipeline (120). The translated ORFs predicted by RAST-Tk were then searched against various databases as follows: diamond blastp versus UniProt (121), hmmscan (http://www.hmmer.org/) versus Pfam (122), EggNOG-mapper v.1.0.1 (123) versus EggNOG (124), hmmscan versus CAZY (125) and MEROPS (126). The results of all annotations combined in one table were loaded as a structured query language (SQL) database and searched with SQL queries. Annotations of large subunits of CO dehydrogenase and catalytic subunits of methane monooxygenase were verified by phylogeny. Briefly, sequences included in the seed alignment of the respective Pfam, together with further sequences with confirmed function or known classification, were obtained from UniProt. Reference and metagenome-derived sequences were aligned to the Pfam alignment with hmmalign (http://www.hmmer.org/), and phylogenetic trees were calculated using FastTree v.2.1.11 (127) starting from a bionj tree (128), using the Le-Gascuel substitution model (129) and gamma likelihood optimization.

The MAGs were taxonomically classified by GTDB-Tk (36) and by the 16S rRNA gene if it was present in a MAG. The GTDB-Tk classification is based on (i) placement in the phylogenomic tree, (ii) relative evolutionary divergence (RED) value as established by Parks et al. (35), and (iii) average nucleotide identity to the closest related genome within a genus. For novel genera, families, and orders, local phylogenetic trees were calculated *de novo* based on GTDB alignment (35) by using FastTree (same settings as described above).

### Functional comparison.

MAGs were grouped using a presence-absence matrix of orthologous proteins as assigned by EggNOG-mapper v.1.0.1 (123, 124). When comparing all MAGs spanning several different phyla, we further summarized different EggNOGs with the same assigned function. MAGs were clustered based on present EggNOGs using the Bray-Curtis dissimilarity matrix and average linkage clustering (vegan package, R) (105).

When the genomic potentials of *Rubrobacteraceae* MAGs and isolate genomes were compared, the EggNOGs were not summarized by function.

The annotations resulting from other sources were checked to confirm the observation and to clarify the function as far as possible.

### Index of replication.

Indices of replication were calculated with iRep (62) using standard settings. Only indices that passed all thresholds and corrections are reported.

### Data availability.

rRNA gene amplicon reads, unassembled metagenomic reads, metagenome assemblies, and annotated MAGs have been deposited in the European Nucleotide Archive under project number PRJEB36534. Bash and R code of all sequence analysis steps can be found at https://github.com/meierdv/avdat_metagenome.
