# Supplementary material for: Distribution of Mixotrophy and Desiccation Survival Mechanisms across Microbial Genomes in an Arid Biological Soil Crust Community
Source: mSystems. 2021 Jan 12;6(1):e00786-20. doi: 10.1128/mSystems.00786-20 (PMC7901476; doi:10.1128/mSystems.00786-20)
Supplement: TABLE S1 [file mSystems.00786-20-st001.pdf]

Table S1: 16S rRNA gene amplicon sequencing metrics

	Total	A01	A09	A17	B09	B14	B20	C01	C06	C10	D03	D10	D15	E03	E12	E20	F01	F08	F19	G02	G08	G19	H01	H10	H19
**Filtered reads**	235952	7249	7037	6857	8757	7918	9735	11633	15575	19687	11115	9642	6449	11729	11171	8737	5871	6768	7222	15518	10909	5048	5236	14053	12036
**# ASVs**	1056	157	176	194	181	181	187	280	289	356	185	220	169	279	229	207	129	147	148	293	207	125	147	276	262
**# SWARM OTUs**	921	155	173	190	176	177	183	274	280	346	178	214	161	271	222	202	126	146	145	282	203	125	146	268	257
**% of reads assigned to 25 ubiquitous OTUs**		63%	48%	41%	47%	48%	52%	48%	58%	52%	59%	44%	37%	41%	60%	51%	68%	56%	64%	42%	58%	59%	54%	43%	48%
**% of reads assigned to 103 OTUs present in 24>12 samples**		25%	32%	35%	34%	32%	29%	22%	21%	21%	17%	23%	23%	31%	23%	30%	22%	29%	23%	32%	27%	32%	31%	33%	28%
**% of reads assigned to 556 OTUs present in 3 and less samples**		2%	6%	7%	6%	6%	6%	7%	5%	8%	5%	8%	11%	7%	4%	4%	2%	7%	4%	5%	3%	3%	4%	6%	10%
