# Supplementary material for: Distribution of Mixotrophy and Desiccation Survival Mechanisms across Microbial Genomes in an Arid Biological Soil Crust Community
Source: mSystems. 2021 Jan 12;6(1):e00786-20. doi: 10.1128/mSystems.00786-20 (PMC7901476; doi:10.1128/mSystems.00786-20)
Supplement: TEXT S1 [file mSystems.00786-20-s0001.pdf]

Distribution of mixotrophy and desiccation survival mechanisms across microbial genomes in an arid biological soil crust community – Supplementary methods

Dimitri V. Meier, Stefanie Imminger, Osnat Gillor, Dagmar Woebken

MAG disclaimer

Metagenome-assembled genomes (MAGs) are collections of contigs considered to belong to the same genome based on multiple agreeing criteria. Some uncertainty of attribution might exist for short contigs, and metagenomic bins can hardly resolve strain diversity. Therefore, we regard the bins as “population genomes”, representing the metabolic potential encoded within a microbial population - cells of the same species occupying a defined niche within the microbial ecosystem. The metabolic potential encoded in the genome sets the limits for potential ecological niches this population could occupy. It further allows for predictions and hypothesis about the activities of different populations and their interactions. The realized niche will depend on interactions with the abiotic environment and other organisms and will be reflected in the fraction of the encoded genomic potential that is transcribed and translated into proteins.

Metagenome analysis

In this section we describe the analysis of the metagenomes in more detail with reference to example scripts with code, which can be found on https://github.com/meierdv/avdat_metagenome. The scripts are numbered in the sequence of execution. Scripts that can be executed simultaneously have the same number. The scripts are rather presented for transparency of the code and parameters used than to be executed as a ready-to-use pipeline. Some parameters are specific to the environment of the Vienna Life Sciences Cluster (modular software management system, SLURM workload manager, databases locations etc.) and might need to be adjusted for differently organized systems. Also the decision whether a "for loop" or an "array" was used often depended on practicalities and free resources on the cluster.

In the beginning we started with a project folder containing the fastq files named by sample names in a subfolder called "reads" and a Sample_list.txt file containing the names of the samples.

In the beginning we start with a project folder containing the fastq files named by sample names in a subfolder called "reads" and a Sample_list.txt file containing the names of the samples.

Trimming and error-correction

Commands in: 01_Trimming_and_error_correction.sh

Raw reads received from the sequencing facility were trimmed using BBduk from the BBtools package (10). Adapters were removed from the "left", 5' end of the reads based on a reference fasta file containing possible variations of Illumina adapters. The first 11 bases from the left end of the reads were trimmed, as well as low quality bases (below q10) from both ends . After the trimming, reads still should be 100 bp or longer and would be discarded otherwise.

The trimmed reads were error-corrected using the Bayes-Hammer error-correction module (11) of the SPAdes assembler (12).

From this step on only the trimmed, error-corrected reads were used for all downstream operations. These are also the reads that were uploaded to the Sequence Read Archive.

Assessing diversity with PhyloFlash

Commands in: 02_PhyloFlash.sh

We used PhyloFlash (13) to assess the diversity of the sequence data. PhyloFlash maps the raw reads to the SILVA database and assembles the reads that mapped into longer SSU rRNA fragments. For generating the figures we used the mapping data, considering taxa as detected when 3 or more reads were mapping to them.

Such assessment of metagenome composition is more advantageous than blast-based classifications of short reads for two reasons:

- First, for blast analysis, fragments of functional genes would be translated to proteins from short reads. As the resulting amino acid sequence would be even shorter than the nucleotide sequence of the fragment, the blast analysis would be based on limited sequence information. For rRNA genes the nucleotide sequence itself is conserved and thus represents a longer sequence information fragment for analysis.

- Second, and maybe most important, is that the rRNA databases still contain a lot more sequence information. Even some described isolates are still lacking publicly available genomes, but their rRNA sequences are deposited in SILVA. Also many uncultured, environmental clades identified via sequences from clone libraries, amplicon libraries etc. are present in the SILVA database. While genomes of entire orders or families are missing, the affiliation of a clade with an environment etc. is already established based on their rRNA gene information. Even more genomes are missing for eukaryotic organisms; thus all reads from fungi, animals and plants might end up being unclassified when simply blasted against NCBI Nr.

Due to these reasons, the community profile will be more precise when reads are mapped with PhyloFlash to SILVA.

Initial assembly

Commands in: 02_Innitial_Megahit_Assembly.sh

This step can be run in parallel with the above-described PhyloFlash operation. Both operations only use the reads.

To reduce the amount of data and confusion created e.g. by uneven coverage of certain genomes due to microdiversity within populations, we first "normalized" the reads with BBnorm (10). In fact, it is rather cutting the maximum coverage down to the desired depth, in our case approx. 42 x (42x read depth = 33x k-mer depth with our mean read length).

The normalized reads from all samples were then used to generate the initial metagenome assembly. To increase the chances for finding a fitting k-mer size for different population with different levels of genomic microdiversity we do iterative assembly in k-mers starting at 21 and up to 137 (which is the average read length after trimming) in small steps of 10. All contigs below 1000 bp are discarded.

Assessing coverage of contigs in different samples

Commands in: 03_BBmap.sh

Assigning of contigs to different genomes is based on 1) their composition (measured in CG-content and tetranucleotide frequencies) and 2) on the pattern of their relative abundance in different samples ("differential coverage"). To obtain the differential coverage information we map the non-normalized (!), error-corrected reads from each sample to the assembly. The identity cut-off is set to 95%, which is the average nucleotide identity (ANI) cut-off for a species.

In order to apply the “differential coverage” approach for binning, we chose samples with different composition based on our 16S rRNA gene analysis (Figure 3). We wanted the populations to vary in abundance across samples, so that they can be more easily distinguished by their differential coverage pattern.

Binning contigs into genomic bins

Commands in: 04_Multi-binning.sh

Now the contigs can be sorted into different bins based on their composition and differential coverage. There are several algorithms available to perform this task and their performance might differ in recognizing different patterns. One binning tool might perform better in recognizing certain patterns and worse recognizing others. Therefore, we ran several of them (MetaBAT: 14, MaxBin: 15, CONCOCT: 16, Metawatt: 17) and then let DAS_Tool (18) pick the best bins and dereplicate the dataset.

Evaluating the initial bins with CheckM

Commands in: 05_CheckM.sh

In the end the contamination and completeness of the bins is assessed based on lineage-specific single-copy genes. To determine which lineage to choose CheckM (19) first places the genomes into a phylogenetic tree. This tree and placement of the genomes can be visualized with e.g. ARB (20) and was used to choose groups of bins for later clade-specific re-assembly based on their phylogeny.

(Evaluating the initial bins with Anvi'O)

Commands in: 05_Anvio_part1.sh, 06_Diamond_vs_NR.sh, 07_Anvio_part2.sh

Anvi'O (21) is a well described interactive tool for metagenome visualization and evaluation (http://merenlab.org/software/anvio/). A lot of different information can be imported, stored and visualized in an Anvi'O database. This becomes more important later and can be skipped at this stage.

However, part of this step is a taxonomic classification of the contigs via a diamond blastp (22) of encoded genes against NCBI-Nr database and then summarazing this information on contig level by Anvi'O.

Re-assembly and new binning

Commands in: 07_Collect_reads_for_reassembly.sh, 08_Reassembly_SPAdes_and_mapping.sh, 09_Multi-binning_of_reassemblies.sh, 10_Anvio_reassembly_part1.sh, 11_Anvi_Profile_reassembly_array.sh, 12_Anvio_reassembly_part2.sh, 13_Anvio_summarize_collection.sh, 14_dRep.sh, 15_GTDB_and_CheckM.sh

Assembling metagenomic data, especially from complex environments like soil, is always challenging due to high diversity of genomes present in the sample. Obviously, metagenomes from environments with lower diversity (enrichment cultures, consortia with few partners, extreme environments like acidic mines offering only few niches) assemble into longer contigs, which are then in turn easier to attribute to the different genomes.

To improve our assembly we therefore aimed to reduce the diversity of the dataset by splitting it up. The idea is to pick out the reads that map only to certain genomes and assemble them again from scratch to achieve a better assembly. This could be done for each of the initial bins, but we were concerned to loose information this way. Especially some small, incomplete bins might be splinters of a larger population genome etc. This was indeed the case with the most abundant genome in the dataset, a Rubrobacter sp., which was initially split up in two bins (complementary in terms of completeness and having no other bins possibly matching in terms of very high coverage).

Therefore, we decided to perform the re-assembly by taxonomic clades, which would still reduce the dataset complexity significantly but would potentially prevent loosing information.

We collected all bins, no matter how complete or contaminated, from a taxonomic group (based on the CheckM tree placement) into a fasta file, which was then used as a reference for "read fishing" (mapping). The reads that mapped to the reference were then assembled de-novo using SPAdes (12). With our computational resources it was challenging to use SPAdes, which we believe is a better assembler, on the whole dataset from the start. But on a reduced volume of data it could be run without allocation of too much RAM or CPU cores.

Acetobacteraceae
Acidimicrobiales
Acidobacteriota
Actinobacteria
Actinomycetales
Armatimonadota
Arthrobacter-related
Bacteroidota
Betaproteobacteriales
Chloroflexota
Cyanobacteria
Dehalococcoidetota
Deinococcota
Frankiales
Gemmatimonadota
Geodermatophilaceae
Nocardioidaceae
Planctomycetota
Propionibacteriaceae
Pseudonocardiaceae
Rhizobiales
Rhodobacteraceae
Rubrobacteraceae
Rubrobacter_01*
Sphingomonadales
Sphingomonas
Thermoleophilia
Thermomicrobiales
Verrucomicrobiaceae

The re-assembly was performed for the following categories:

*single most abundant *Rubrobacteraceae* genome assembled separately.

The binning was repeated for the newly generated assemblies and the bins were inspected in Anvi'O. This step is probably the most arbitrary one. The anvi-refine interactive interface of Anvi'O visualizes the contigs of a bin in a dendrogram, clustered by a combination of tetranucleotide frequencies and differential coverage pattern. It also displays the taxonomic classification of the contigs and locations of single-copy genes. In this interface contigs can be selected and assigned to a new derived bin, excluding the other not selected contigs. We checked the bins for contigs that

would strikingly cluster apart showing a different coverage pattern than the rest of the bin. Usually these were clusters of short contigs, which were probably too short for confident assignment.

While removing such contigs in an interactive interface might introduce human bias, ultimately it generates more conservative bins since we are only removing contigs from automatically generated bins. In our study we tried to focus on the presence of genes in the generated bins (or MAGs). However, for certain absent genes such as encoding nitrogen fixation or respiratory nitrate reduction we also searched the unbinned fraction and for nifH mapped the raw reads to a nifH database to make sure we did not loose them in our assembly and binning process.

New vs. old bin set

We compared the bin sets fulfilling the criteria of over 50% completeness and less than 10% contamination after the initial bulk assembly and after the re-assembly. We also performed clustering by Min-HASH distances and ANI in dRep (23) to find corresponding bins in old and new set. After the re-assembly step, we gained 20 bins that were not among the bins with > 50% completeness and < 10% contamination after the first assembly. We lost 8 bins that were in this category after the first assembly, so we added them again to the final set.

Most bins were generated in both assembly steps and most had better metrics after the re-assembly. However, we kept in total 16 bins from the initial assembly, either because they were lost in the re-assembly process or because they had better metrics in the old assembly.

	**Old bins**	**New bins**	**Improvement**
Total size (bp)	258,296,985	344,481,516	33%
Contigs	49,904	45,778	9.0%
Contig length N50	10,642	20,936	97%
Average completeness (%)	79	83	6%
Average contamination (%)	3	3	1%
Bins with SSU sequence	38	52	37%

Taxonomic classification of the bins

Commands in: 15_GTDB_and_CheckM.sh, 16_FastTree_slurm.sh

The bins were taxonomically classified using the GTDB toolkit (24), which bases its analysis on 120 translated marker genes. If the MAGs are close enough to a reference genome to potentially belong to the same species, GTDB-Tk provides an ANI value. Additionally it provides a "relative evolutionary distance" (RED) value, which can be compared to the RED values calculated for known taxonomic groups during GTDB creation (8). All of the generated MAGs represented new species, or more precise, species not contained in GTDB. Many were only given family-level classification. For those, we briefly calculated new phylogenetic trees to verify their placement.

The concatenated amino-acid alignment of 120 translated marker genes used by GTDB was loaded into ARB. This alignment contains GTDB reference genomes as well as our MAGs. Additionally, we loaded the GTDB tree to this alignment. In the tree we marked the phylogenetic clades to be recalculated and the nearest bigger clade not belonging to the target group was used as an outgroup. The alignment was filtered by conservation. Only positions conserved in over 25% of sequences and containing less than 50% gaps were used. These filtered alignments were exported from ARB as gap-containing fasta files and used as input for FastTree (25). The calculated trees were imported back and visualized in ARB.

Based on these new trees we verified the classification of bins initially given by GTDB-Tk. We mainly checked if bins supposed to belong to e.g. a new family clustered clearly separate from other defined families and if the branch lengths looked comparable to those of other defined families.

Functional annotation

Commands in: 16_InterPro_array.sh, 16_Emapper_array.sh, 16_Diamond_vs_Uniprot.sh, 16_HMM_dbCAN_array.sh, SQL_log.sql, 17_Counting_Functions.R

Assigned annotations, including the individual results of different annotation tools in: Suppl_File01_Searched_functions.xlsx

For gene prediction we uploaded the genomes to RAST (not MG-RAST, but RAST as separate genomes) and ran the RAST-ToolKit pipeline (26), which predicts ORFs as a consensus from three gene-calling tools (Prodigal: 19, Glimmer 3: 20, GeneMark: 21). The results were downloaded in gff3 format, reformatted and imported into the Anvi'O database.

When searching encoded functionalities we decided not to rely on general functional categories as many bulk-metagenome studies do for two reasons:

1) Many of these categories are too general and are expected to be present in all organisms, e.g. "DNA metabolism".

2) The systems of categories and pathways databases such as KEGG pathways (30) or SEED subsystems (31) were designed to check completeness of pathways in a given organism and make metabolic predictions or perform metabolic modeling for this microorganism. It means that certain enzymes with fairly general function will be assigned to several pathways. When using such pathways database to screen metagenomes and counting the hits for each pathway, such general enzymes will contribute hits to several pathways/categories without being indicative of the pathway presence.

Therefore, we decided to focus on genes encoding key enzymes enabling a pathway/metabolism, rather than on genes involved in pathways yet having a more general function and thus, if found without the key enzyme, are not suitable as definitive indicators.

Our annotation derives from several sources which were all combined in one big table containing all the results for each gene. The table was generated by merging the tabular outputs of different sources based on gene id as unique identifier. Each source might be better in annotating some genes and worse in annotating others. We searched the table by text string search via SQL queries, extending the search iteratively with following logic:

1. If there is a known Pfam (32) domain definitive for a function we would first search for this domain. If not, we would search for EC numbers or key words in the RAST annotation. We would inspect the hits for presence of necessary Pfam domains.

2. We would check the EggNOG (33) id of the hits and do the search also including genes with the same EggNOG id, even if not with the same RAST annotation. EggNOG ids are very valuable since they assign a gene to a group of orthologs without the necessity of knowing its function. Therefore, once the EggNOG annotation is done, one can look for the same id to find orthologs instead of performing a new sequence similarity search. Of course, this is still limited to the ortholog groups in the EggNOG database. Yet, the database is much larger than COG (34) (and it includes the COG othologous groups as well), because the ortholog groups don't need functional annotation and are generated in an automated unsupervised manner from public databases.

3. Uniprot (35) was used for verification.

4. For carbohydrate-active enzymes (CAZYmes), the results of HMM search vs CAZY database (36) were used as the only source, since none of the other annotation source recognizes and resolves the variety of CAZYmes as good as this dedicated resource.

5. The search results for each metabolism or pathway, basically for each category in Fig, 3/S3, were exported as tables and checked manually. Usually the RAST annotation was copied over to genes having same EggNOG and Pfam motif, yet not having any RAST annotation.

Here is an example of a hydrogenase search query intended to find all type of hydrogenases and exclude unrelated enzymes containing the string "hydrogenase" such as de**hydrogenase**. Utilized SQL queries can be found in SQL_log.sql file.

```
SELECT * from Avdat_crust_all_annotations

WHERE RAST like '% hydrogenase%subunit%' AND Pfam like '% hydrogenase%' OR

Pfam like '%Fe% hydrogenase%' OR

Pfam like '%Nickel% hydrogenase%' OR

Uniprot like 'Hydrogenase%subunit%' AND Pfam like '% hydrogenase%' OR

Uniprot like '%Fe%hydrogenase%subunit%' AND Pfam like '% hydrogenase%'
```

These tables with SQL query results for each metabolism were summarized counting the differently annotated genes per bin. For such categories as transporters or glycosyl-hydrolases the number of differently annotated genes provide an estimate of the substrate range. For pathways, or multi-unit enzymes it provides an estimate of pathway completeness. For example, it is a difference if only a membrane anchor subunit of an enzyme is present or also the two large catalytic subunits. In a way this way of counting also reduces the possible bias of binning. Bins might contain contigs from very closely related strains (what checkM calls "strain heterogeneity") and therefore duplications of certain genes which could be artifacts of binning. Therefore, we found counting number of different annotations more meaningful than summing up all hits.

The Supplementary file 1 is a table showing all the genes searched during our analysis and their original annotations from different sources.

Clustering of genomes based on EggNOG orthologs

As mentioned above, assignment of EggNOG id might be a valuable shortcut for comparative genome analysis and high level EggNOG groups (domain level, which we used) could also allow for functional comparison of more distant genomes. We went even a step further and summarized different EggNOGs with the same function assigned. These categories were used to generate a dissimilarity matrix of MAGs based on presence-absence of functions (EggNOGs). The matrix turned out to be large and sparse (containing many zeroes). Since the absence of a function in a MAG is not very meaningful, due to possible MAG incompleteness, we used the Bray-Curtis index for calculating dissimilarity. Clustering of MAGs based on this matrix showed that most clustered according to their taxonomic groups, which was expected (all *Cyanobacteria* are phototrophic, to name the most obvious correlation of taxonomy and function). The exception were the *Thermoleophilia* and *Rubrobacteria* which clustered apart from other *Actinobacteria* and together with *Chloroflexi*. This demonstrates the functional diversity of members within the *Actinobacteria* phylum and that phylum-level generalizations can be misleading.

Supplementary References:

1. McLaren MR, Willis AD, Callahan BJ. 2019. Consistent and correctable bias in metagenomic sequencing experiments. Elife 8:e46923.

2. Zahradka K, Slade D, Bailone A, Sommer S, Averbeck D, Petranovic M, Lindner AB, Radman M. 2006. Reassembly of shattered chromosomes in Deinococcus radiodurans. Nature 443:569–573.

3. Egas C, Barroso C, Froufe HJC, Pacheco J, Albuquerque L, da Costa MS. 2014. Complete genome sequence of the Radiation-Resistant bacterium Rubrobacter radiotolerans RSPS-4. Stand Genomic Sci 9:1062–1075.

4. Griese M, Lange C, Soppa J. 2011. Ploidy in cyanobacteria. FEMS Microbiol Lett 323:124–131.

5. Wear EK, Wilbanks EG, Nelson CE, Carlson CA. 2018. Primer selection impacts specific population abundances but not community dynamics in a monthly time-series 16S rRNA gene amplicon analysis of coastal marine bacterioplankton. Environ Microbiol 20:2709–2726.

6. Steven B, Gallegos-Graves LV, Starkenburg SR, Chain PS, Kuske CR. 2012. Targeted and shotgun metagenomic approaches provide different descriptions of dryland soil microbial communities in a manipulated field study. Environ Microbiol Rep 4:248–256.

7. Li JY, Jin XY, Zhang XC, Chen L, Liu JL, Zhang HM, Zhang X, Zhang YF, Zhao JH, Ma ZS, Jin D. 2020. Comparative metagenomics of two distinct biological soil crusts in the Tengger Desert, China. Soil Biol Biochem 140:107637.

8. Parks DH, Chuvochina M, Waite DW, Rinke C, Skarshewski A, Chaumeil PA, Hugenholtz P. 2018. A standardized bacterial taxonomy based on genome phylogeny substantially revises the tree of life. Nat Biotechnol 36:996.

9. King GM, Weber CF. 2007. Distribution, diversity and ecology of aerobic CO-oxidizing bacteria. Nat Rev Microbiol 5:107–118.

10. Brian Bushnell. 2017. BBtools. https://sourceforge.net/projects/bbmap/

11. Nikolenko SI, Korobeynikov AI, Alekseyev MA. 2013. BayesHammer: Bayesian clustering for error correction in single-cell sequencing. BMC Genomics 14:S7.

12. Bankevich A, Nurk S, Antipov D, Gurevich AA, Dvorkin M, Kulikov AS, Lesin VM, Nikolenko SI, Pham S, Prjibelski AD, Pyshkin A V, Sirotkin A V, Vyahhi N, Tesler G, Alekseyev MA, Pevzner PA. 2012. SPAdes: a new genome assembly algorithm and its applications to single-cell sequencing. J Comput Biol2012/04/18. 19:455–477.

13. Gruber-Vodicka HR, Pruesse E, Seah Brandon K. 2017. phyloFlash. https://github.com/HRGV/phyloFlash/blob/master/docs/index.md

14. Kang DD, Froula J, Egan R, Wang Z. 2015. MetaBAT, an efficient tool for accurately reconstructing single genomes from complex microbial communities. PeerJ2015/09/04. 3:e1165.

15. Wu YW, Tang YH, Tringe SG, Simmons BA, Singer SW. 2014. MaxBin: An automated binning method to recover individual genomes from metagenomes using an expectation-maximization algorithm. Microbiome 2:26.

16. Alneberg J, Bjarnason BS, de Bruijn I, Schirmer M, Quick J, Ijaz UZ, Lahti L, Loman NJ, Andersson AF, Quince C. 2014. Binning metagenomic contigs by coverage and composition. Nat Methods2014/09/15. 11:1144–1146.

17. Strous M, Kraft B, Bisdorf R, Tegetmeyer HE. 2012. The binning of metagenomic contigs for microbial physiology of mixed cultures. Front Microbiol2012/12/12. 3:410.

18. Sieber CMK, Probst AJ, Sharrar A, Thomas BC, Hess M, Tringe SG, Banfield JF. 2018. Recovery of genomes from metagenomes via a dereplication, aggregation and scoring strategy. Nat Microbiol 3:836–843.

19. Parks DH, Imelfort M, Skennerton CT, Hugenholz P, Tyson GW, Hugenholtz P, Tyson GW. 2015. CheckM: assessing the quality of microbial genomes recovered from isolates, single cells, and metagenomes. Genome Res2015/05/16. 25:1043–1055.

20. Ludwig W, Strunk O, Westram R, Richter L, Meier H, Yadhukumar, Buchner A, Lai T, Steppi S, Jobb G, Forster W, Brettske I, Gerber S, Ginhart AW, Gross O, Grumann S, Hermann S, Jost R, Konig A, Liss T, Lussmann R, May M, Nonhoff B, Reichel B, Strehlow R, Stamatakis A, Stuckmann N, Vilbig A, Lenke M, Ludwig T, Bode A, Schleifer KH. 2004. ARB: a software environment for sequence data. Nucleic Acids Res2004/02/27. 32:1363–1371.

21. Eren AM, Esen OC, Quince C, Vineis JH, Morrison HG, Sogin ML, Delmont TO. 2015. Anvi’o: an advanced analysis and visualization platform for ’omics data. PeerJ2015/10/27. 3:e1319.

22. Buchfink B, Xie C, Huson DH. 2015. Fast and sensitive protein alignment using DIAMOND. Nat Methods 12:59–60.

23. Olm MR, Brown CT, Brooks B, Banfield JF. 2017. dRep: a tool for fast and accurate genomic comparisons that enables improved genome recovery from metagenomes through de-replication. ISME J 11:2864–2868.

24. Chaumeil P-A, Mussig AJ, Hugenholtz P, Parks DH. 2019. GTDB-Tk: a toolkit to classify genomes with the Genome Taxonomy Database. Bioinformatics.

25. Price MN, Dehal PS, Arkin AP. 2010. FastTree 2 - approximately maximum-likelihood trees for large alignments. PLoS One2010/03/13. 5:e9490.

26. Brettin T, Davis JJ, Disz T, Edwards RA, Gerdes S, Olsen GJ, Olson R, Overbeek R, Parrello B, Pusch GD, Shukla M, Thomason JA, Stevens R, Vonstein V, Wattam AR, Xia F, Xia F. 2015. RASTtk: a modular and extensible implementation of the RAST algorithm for building custom annotation pipelines and annotating batches of genomes. Sci Rep 5:8365.

27. Hyatt D, Chen GL, Locascio PF, Land ML, Larimer FW, Hauser LJ. 2010. Prodigal: prokaryotic gene recognition and translation initiation site identification. BMC Bioinformatics2010/03/10. 11:119.

28. Kelley DR, Liu B, Delcher AL, Pop M, Salzberg SL. 2012. Gene prediction with Glimmer for metagenomic sequences augmented by classification and clustering. Nucleic Acids Res2011/11/22. 40:e9.

29. Borodovsky M, McIninch J. 1993. GENMARK: Parallel gene recognition for both DNA strands. Comput Chem 17:123–133.

30. Kanehisa M, Goto S. 2000. KEGG: Kyoto encyclopedia of genes and genomes. Nucleic Acids Res1999/12/11. 28:27–30.

31. Aziz RK, Bartels D, Best AA, DeJongh M, Disz T, Edwards RA, Formsma K, Gerdes S, Glass EM, Kubal M, Meyer F, Olsen GJ, Olson R, Osterman AL, Overbeek RA, McNeil LK, Paarmann D, Paczian T, Parrello B, Pusch GD, Reich C, Stevens R, Vassieva O, Vonstein V, Wilke A, Zagnitko O. 2008. The RAST Server: rapid annotations using subsystems technology. BMC Genomics2008/02/12. 9:75.

32. Finn RD, Bateman A, Clements J, Coggill P, Eberhardt RY, Eddy SR, Heger A, Hetherington K, Holm L, Mistry J, Sonnhammer EL, Tate J, Punta M. 2014. Pfam: the protein families database. Nucleic Acids Res2013/11/30. 42:D222-30.

33. Huerta-Cepas J, Szklarczyk D, Forslund K, Cook H, Heller D, Walter MC, Rattei T, Mende DR, Sunagawa S, Kuhn M, Jensen LJ, von Mering C, Bork P. 2016. eggNOG 4.5: a hierarchical orthology framework with improved functional annotations for eukaryotic, prokaryotic and viral sequences. Nucleic Acids Res 44:D286–D293.

34. Tatusov RL. 2000. The COG database: a tool for genome-scale analysis of protein functions and evolution. Nucleic Acids Res 28:33–36.

35. Magrane M, Consortium U. 2011. UniProt Knowledgebase: a hub of integrated protein data. Database2011/03/31. 2011:bar009.

36. Lombard V, Golaconda RH, Drula E, Coutinho PM, Henrissat B. 2014. The carbohydrate-active enzyme database (CAZy) in 2013. Nucl Acids Res 42.
