# Supplementary material for: Distribution of Mixotrophy and Desiccation Survival Mechanisms across Microbial Genomes in an Arid Biological Soil Crust Community
Source: mSystems. 2021 Jan 12;6(1):e00786-20. doi: 10.1128/mSystems.00786-20 (PMC7901476; doi:10.1128/mSystems.00786-20)
Supplement: TEXT S2 [file mSystems.00786-20-s0002.pdf]

Distribution of mixotrophy and desiccation survival mechanisms across microbial genomes in an arid biological soil crust community – Supplementary discussion

Dimitri V. Meier, Stefanie Imminger, Osnat Gillor, Dagmar Woebken

Differences between amplicon and metagenome data

The analysis of microbial community composition based on sequence data is subject to several biases at the different steps of sample preparation or data analysis (summarized in 1). The microbial community composition in the soil crusts based on extracted DNA was analyzed and presented in three different ways in this study (Fig. 3): 16S rRNA gene amplicon sequencing, mapping of unassembled metagenomic reads to the SILVA database, and relative abundance of metagenome-assembled genomes (MAGs) based on their read coverage. The relative abundances of some microbial taxa differed considerably between the different analyses. In the following, we discuss the possible reasons for this discrepancy.

DNA extraction bias:

All analyses for one crust sample are based on the same DNA extract. Therefore, we can exclude the possibility that different extractions led to the observed discrepancy.

Metagenome assembly and binning bias

The taxa missing among the MAGs are, for example, *Archaea* and *Firmicutes*. This could be explained by their low relative abundance in the metagenomic data (visible via the mapping of unassembled metagenomic reads to the SILVA database, Figure 2, Suppl. Dataset 02), which might have precluded assembly and binning. However, we were successful in obtaining MAGs of four cyanobacterial taxa, despite the lower cyanobacterial proportion in the unassembled reads in comparison to the amplicon data (Figure 2).

Discrepancy between biomass and genome copies among community members:

The possibility exists that cyanobacterial abundance in the metagenome reflects their true genome abundance in the sample, which is lower than expected based on e.g. visual observations (Fig. 1E) that reflects their large biomass in BSCs. First, cyanobacterial cells are many times larger than cells of other community members, therefore they can constitute a high proportion of the total biomass, while having low cell numbers. Second, organisms like *Rubrobacter* might rely on genome repair via multiple genome copies, similar to mechanisms known from *Deinococcus radiodurans* (2, 3) and thus they might contribute a disproportionately high number of genomes to the overall metagenome. Although cyanobacteria might as well contain several genome copies per cell (4), the ratio of genome per biomass might be much higher among e.g. *Actinobacteria* than among *Cyanobacteria*. This could lead to cyanobacterial genomes showing low relative abundance in the metagenome despite constituting a large proportion of biomass in the crusts.

Primer-based PCR bias

The fact that cyanobacteria reads are of higher abundance in the 16S rRNA gene amplicon sequencing data than in the metagenome data generated from the same DNA extract and also classified with the SILVA database, points towards additional influencing factors. Choice of PCR primers is a well known source of bias when analyzing microbial community composition based on amplified marker genes (1) and countless studies comparing different primer pairs and their coverage of the microbial taxa exist. In a study testing different primer pairs on aquatic microbial community and comparing the determined community composition to unassembled metagenomic data the primer pair 341f/785R was shown to significantly overestimate *Cyanobacteria* (5). However, our primer pair of choice (referred to as 515F-Y and 806RB by Wear *et al.*) was not shown to have a bias regarding cyanobacterial sequences when compared to metagenomic data (5). Yet, the comparison was performed on aquatic communities containing completely different cyanobacteria than BSC. It cannot be ruled out that the primer pair used by us to amplify the 16S rRNA genes specifically overestimates the relative abundance of *Microcoleus* cyanobacteria.

Together with the above mentioned relationships between biomass, cell numbers and genome copies per cell, such positive primer bias would be the only explanation for the observed discrepancy.

Previous observation of low cyanobacteria abundance in other BSC metagenomic studies

Low abundance of cyanobacteria in metagenomes from BSCs was previously observed in BSCs from Nevada Desert, USA (6) and Tengger Desert, China (7). However, in these studies metagenomic sequences were classified based on BLAST hits in NCBI-Nr database and not on mapping to SILVA database. As suggested by Steven *et al.* (6), the bias might therefore stem from under-representation of *Cyanobacteria* by genomes in the NCBI Nr database.
