# Supplementary material for: Distribution of Mixotrophy and Desiccation Survival Mechanisms across Microbial Genomes in an Arid Biological Soil Crust Community
Source: mSystems. 2021 Jan 12;6(1):e00786-20. doi: 10.1128/mSystems.00786-20 (PMC7901476; doi:10.1128/mSystems.00786-20)
Supplement: TABLE S2 [file mSystems.00786-20-st002.pdf]

Table S2A: Metagenomic sequencing efforts and representation of raw reads in the assemblies and MAGs

Sample	Produced data (Gbp)	Mio. raw read pairs (2x150 bp)	Mio. Error-corrected Read pairs	Nonpareil % of diversity covered	Nonpareil diversity index	Mapping to assembly (with > 95% identity)	Mapping to Final MAGs (with > 95% identity)
**B20**	28	92	84	74%	20.7	54%	36%
**F08**	32	107	96	67%	21.7	40%	24%
**G19**	28	94	85	65%	21.6	43%	31%
**C15-24h**	16	55	52	56%	21.8	34%	26%
**F12-24h**	20	68	66	58%	21.9	34%	24%

Table S2B: Initial metagenome assembly metrics

	Initial assembly (contigs > 1000 bp)
**Total size (Gbp)**	1.14
**Number of contigs**	431,850
**Largest contig (bp)**	167,554
**Contig length N50**	3140
**Contig length L50**	89605
**GC content**	69%
**Mapping B20 (>95% id)**	54%
**Mapping F08 (>95% id)**	40%
**Mapping G19 (>95% id)**	43%
**Mapping C15 (>95% id)**	34%
**Mapping F12 (>95% id)**	34%
