# Supplementary figures and images for: Distribution of Mixotrophy and Desiccation Survival Mechanisms across Microbial Genomes in an Arid Biological Soil Crust Community
Source: mSystems. 2021 Jan 12;6(1):e00786-20. doi: 10.1128/mSystems.00786-20 (PMC7901476; doi:10.1128/mSystems.00786-20)

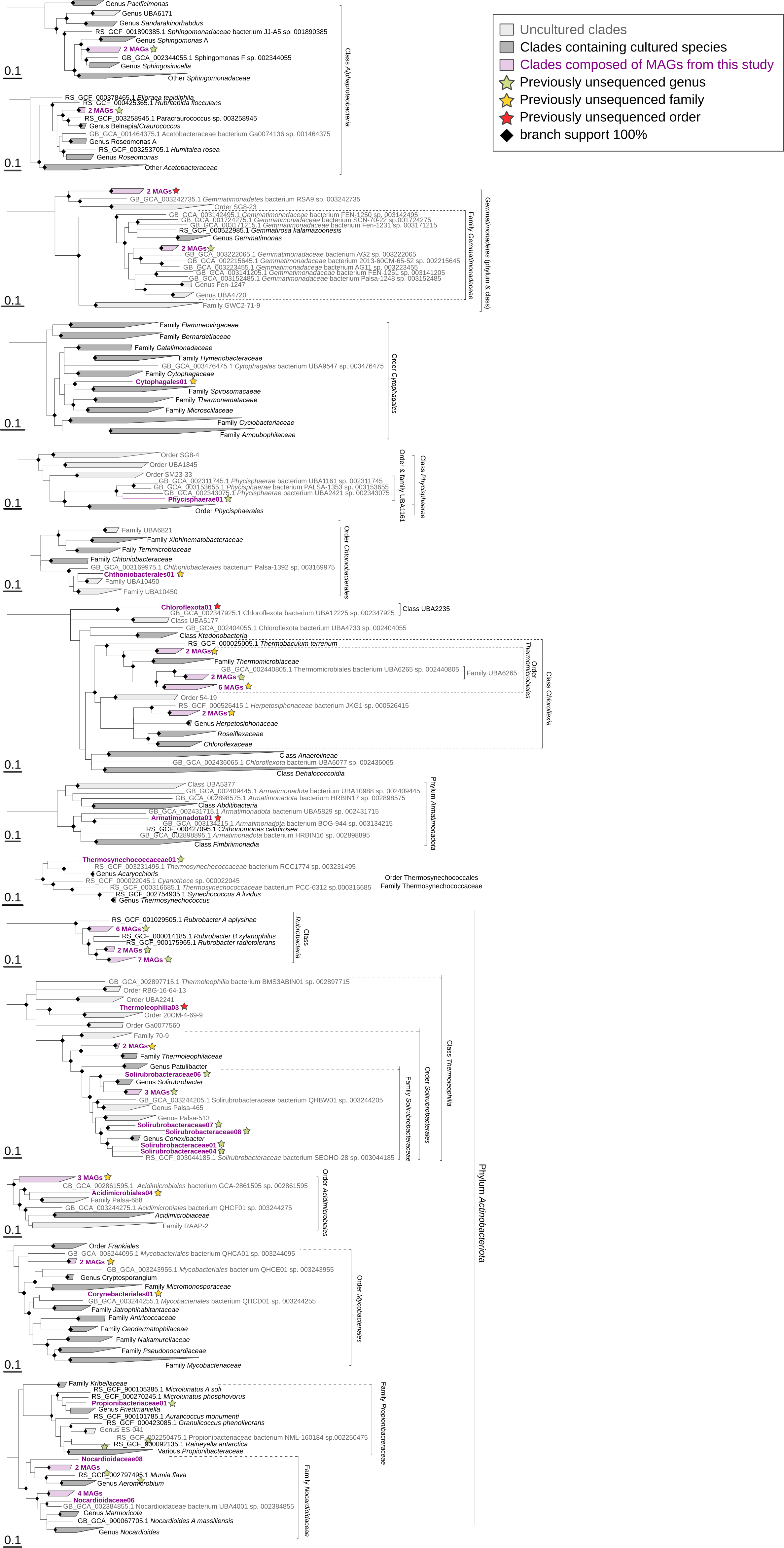

Supplement: FIG S1 [file mSystems.00786-20-sf001.tif]

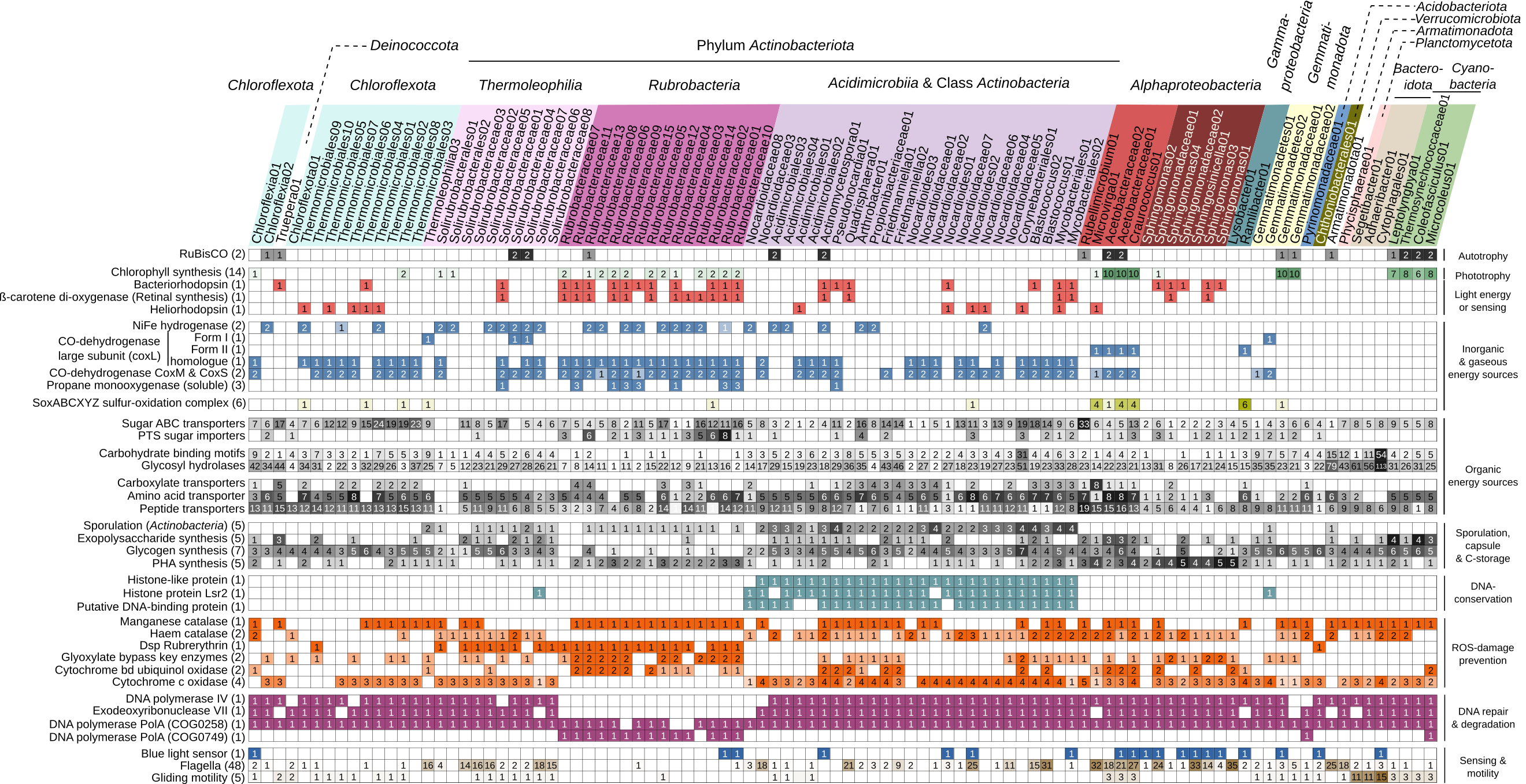

Supplement: FIG S2 [file mSystems.00786-20-sf002.tif]

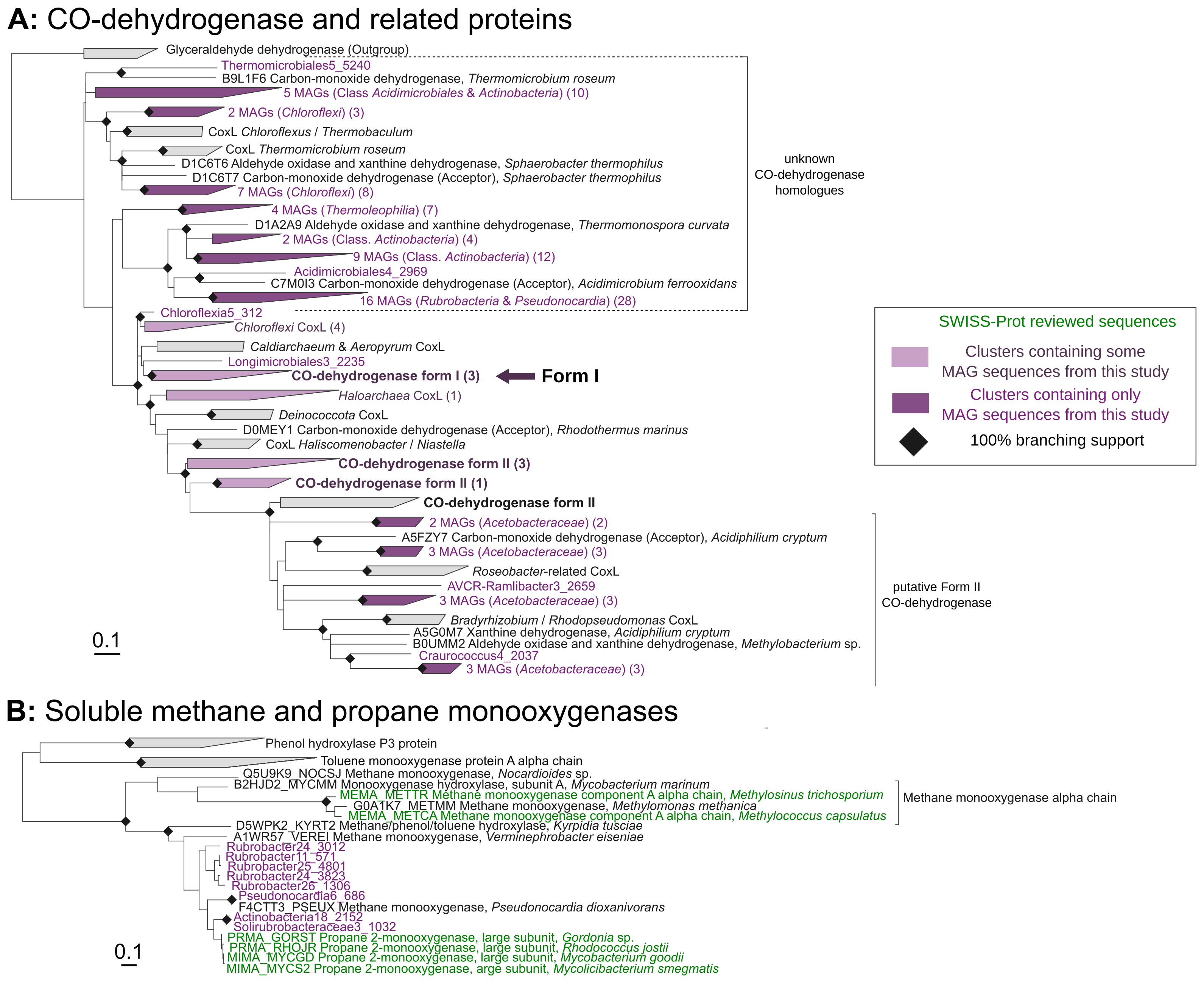

Supplement: FIG S3 [file mSystems.00786-20-sf003.tif]
